# Dietary *Bacillus* spp*.* enhanced growth and disease resistance of weaned pigs by modulating intestinal microbiota and systemic immunity

**DOI:** 10.1186/s40104-020-00498-3

**Published:** 2020-09-15

**Authors:** Yijie He, Cynthia Jinno, Kwangwook Kim, Zhaohai Wu, Bie Tan, Xunde Li, Rose Whelan, Yanhong Liu

**Affiliations:** 1 Department of Animal Science, University of California, Davis, CA 95616 USA; 2 Institute of Animal Science, Chinese Academy of Agricultural Sciences , Beijing, 100193 China; 3 Key Laboratory of Agro-ecological Processes in Subtropical Region, Institute of Subtropical Agriculture, Chinese Academy of Sciences, Changsha, 410125 China; 4 School of Veterinary Medicine, University of California, Davis, CA 95616 USA; 5 Evonik Nutrition & Care GmbH, 63457 Hanau-Wolfgang, Germany

**Keywords:** *Bacillus* spp*.*, Diarrhea, *Escherichia coli*, Gut health, Weaned pigs

## Abstract

**Background:**

Previous research has shown that dietary supplementation of *Bacillus* spp. probiotics exerts beneficial effects on animals’ growth. However, limited studies have evaluated the efficacy of *Bacillus* spp*.* on weaned pigs and their effects on host gut health and microbiome, and systemic immunity using a disease challenge model. The objective of this experiment was to investigate the effects of two *Bacillus* spp*.* strains (*Bacillus subtilis* DSM 32540 and *Bacillus pumilus* DSM 32539) on growth performance, diarrhea, intestinal health, microbiome, and systemic immunity of weaned pigs experimentally infected with an enterotoxigenic *Escherichia coli* (ETEC).

**Results:**

Pigs in PRO1 (*Bacillus subtilis* DSM 32540) had greater (*P* < 0.05) body weight on d 7 and 14 PI, greater (*P* < 0.05) ADG from d 0 to 7 and d 7 to 14 PI, compared with pigs in CON (Control). Pigs in PRO1 had milder (*P* < 0.05) diarrhea on d 2 and 3 PI compared with pigs in CON. However, no differences were observed in growth performance and diarrhea score between PRO2 (*Bacillus pumilus* DSM 32539) and CON groups. Supplementation of PRO1 decreased (*P* < 0.05) lymphocyte counts on d 7 and 14 PI, compared with CON. Supplementation of PRO1 and PRO2 both reduced (*P* < 0.05) total coliforms in mesenteric lymph nodes on d 21 PI. Pigs in PRO2 had greater (*P* < 0.05) goblet cell number and sulfomucin percentage in duodenal villi and greater (*P* < 0.05) sialomucin percentage in jejunal villi than pigs in CON. Supplementation of PRO1 up-regulated (*P* < 0.05) *MUC2* gene expression in jejunal mucosa and reduced (*P* < 0.05) *PTGS-2* and *IL1B* gene expression in ileal mucosa on d 21 PI, compared with CON. Pigs in PRO1 had reduced (*P* < 0.05) relative abundance of families Lachnospiraceae, Peptostreptococcaceae and Pasteurellaceae in the ileum.

**Conclusions:**

Supplementation of *Bacillus subtilis* DSM 32540 improved growth performance, alleviated diarrhea severity, enhanced gut health, and reduced systemic inflammation of weaned pigs infected with ETEC F18. Although *Bacillus pumilus* DSM 32539 was able to alleviate systemic inflammation, it had limited impacts on growth performance and severity of diarrhea of ETEC F18 challenged weaned pigs.

## Background

Post-weaning diarrhea is a commonly occurring and economically important disease in the swine industry worldwide [1]. The disease is mainly caused by an *Escherichia coli* (*E. coli*) pathotype referred to as enterotoxigenic *E. coli* (ETEC), and is characterized by watery diarrhea, dehydration and impaired growth [2]. In the United States, *E. coli* caused post-weaning diarrhea has affected approximately 32.1% to 45.5% of the medium-sized farms from 2000 to 2012, representing huge economic losses to the producers [3–5]. Traditionally, in-feed antibiotics were routinely added to swine diet as prophylactic treatment at times of stress such as post weaning period. However, the frequent use of these in-feed antibiotics in livestock production has driven the selection for antibiotics-resistant microorganisms [6]. The emergence of pathogens that are resistant to medically important antibiotics in humans raises great concerns [7]. In 2017, through the revision of the veterinary feed directory, the Food and Drug Administration (FDA) mandated the removal of in-feed addition of antibiotics for growth promotion purpose in animal production [8]. In order to ensure animal welfare and maintain animal productivity, it is necessary to develop and investigate alternatives to antibiotics.

Probiotics are defined as live microorganisms which, when administered in adequate amounts, confer a health benefit on the host [9]. Some of the widely used probiotics supplements in livestock and poultry production are *Saccharomyces* spp*.*, *Lactobacillus* spp*.*, *Enterococcus* spp., and *Bacillus* spp*.* [10]. *Bacillus* spp*.* probiotics are suitable feed additives due to their ability to form spores that enable them to endure harsh environmental conditions and to germinate in the gut of animals when exposed to adequate nutrients [11]. Previous research has demonstrated that supplementation of probiotic *Bacillus* strains could improve growth performance, reduce incidence of diarrhea, and improve gut morphology in weaned pigs [12]. The potential mechanisms of action include but may not limit to the lists below. 1) *Bacillus subtilis* may modulate the host immune responses by regulating the expression of major cytokines that are involved in initiating and regulating immune responses [13]. 2) *Bacillus subtilis* may also indirectly enhance the expression of tight junction proteins [14]. 3) Moreover, through the production of antimicrobials, *Bacillus subtilis* may affect the composition and function of microbial communities, promoting the growth of beneficial microbes and overall gut health [15, 16]. However, limited studies have evaluated the efficacy of *Bacillus* spp*.* probiotic supplementation on weaned pigs and the effects on host gut health and microbiome, and systemic immunity using an ETEC F18 challenge model. F18-positive ETEC is one of the most dominant strains of ETEC that is responsible for around 33.9% of post-weaning diarrhea in weanling pigs [17]. Therefore, the objective of this experiment was to determine the impacts of a new probiotic strain *Bacillus subtilis* DSM 32540 (WO 2019/002471 A1) compared to an untreated control or *Bacillus pumilus* DSM 32539 (WO 2019/002476 A1) on growth performance, incidence and severity of diarrhea, intestinal health and systemic immunity of weaned pigs experimentally infected with ETEC F18. The intestinal microbial profile was also analyzed to investigate the impacts of probiotics supplementation on intestinal microbiome of weaned pigs.

## Materials and methods

### Animal, housing, experimental design and diet

At approximately 21 d of age, a total of 36 piglets with 15 gilts and 21 barrows [7.61 ± 0.40 kg BW (body weight)] were selected from the Swine Teaching and Research Center of UC Davis and used in this experiment. The 4 sows (multiparous with the parity from 2 to 4) and piglets used in this experiment did not receive *E. coli* vaccines, antibiotic injections, or antibiotics in creep feed. Before weaning, fecal samples were collected from sows and all their piglets destined for this experiment to verify the absence of β-hemolytic ETEC. The ETEC F18 receptor status in all piglets were also tested based on the methods described previously in Kreuzer et al. [18]. All pigs used in this experiment were genotypically susceptible to ETEC F18 infection and free of ETEC F18.

After weaning (around 21 d of age), all pigs were transferred to the Cole facility of UC Davis and were housed in individual pens (0.61 m × 1.22 m) for 28 d, including 7 d before and 21 d after the first ETEC challenge. All pigs had free access to feed and water. Animal rooms were equipped with fans and heaters to achieve the desired thermoneutral zone for nursery pigs. The light period was provided for 12 h starting from 07:30 h.

Pigs were randomly assigned to one of three experimental treatments in a randomized complete block design with pigs’ body weight within sex as the blocking factor and 12 replicates per treatment. The 3 dietary treatments included: 1) the complex nursery basal diet (CON), and 2) inclusion of 500 mg/kg (1 × 10^9^ CFU/kg) of the probiotic *Bacillus subtilis* DSM 32540 (PRO1), or 3) inclusion of 500 mg/kg (1 × 10^9^ CFU/kg) of *Bacillus pumilus* DSM 32539 (PRO2) in the nursery basal diet, respectively. *Bacillus pumilus* DSM 32539 was used as a reference strain due to it not being specifically targeted for *E. coli* pathogens based on unpublished *in vitro* data. Spray-dried plasma, antibiotics, and high levels of zinc oxide exceeding recommendation were not included in the diets. The basal diets were formulated to meet or exceed estimates of nutrient requirements of weaned pigs (Table 1) [19]. The experimental diets were fed to pigs immediately after weaning as a 2-phase feeding program with weeks 1 and 2 as phase 1 and weeks 3 and 4 as phase 2. All diets were provided in mash form.

**Table 1:** Ingredient compositions of experimental diets^a^

Ingredient, %	Control, phase I	Control, phase II
Corn	42.40	48.40
Dried whey	15.00	10.00
Soybean meal	20.00	24.00
Fish meal	4.00	3.00
Barley	10.00	10.00
Soy protein concentrate	3.00	–
Soybean oil	2.10	1.30
Limestone	1.10	1.10
Monocalcium phosphate	0.50	0.45
*L*-Lysine·HCl	0.49	0.46
*DL*-Methionine	0.26	0.21
*L*-Threonine	0.22	0.20
*L*-Valine	0.09	0.08
Salt	0.14	0.10
Vit-mineral^b^	0.40	0.40
Total	100.00	100.00
Calculated energy and nutrient		
Metabolizable energy, kcal/kg	3364	3310
Net energy, kcal/kg	2526	2480
Crude protein, %	20.54	19.77
Arg,^c^%	1.14	1.11
His,^c^%	0.47	0.46
Ile,^c^%	0.76	0.72
Leu,^c^%	1.50	1.44
Lys,^c^%	1.42	1.32
Met,^c^%	0.56	0.50
Thr,^c^%	0.89	0.83
Trp,^c^%	0.31	0.29
Val,^c^%	0.97	0.89
Met + Cys,^c^%	0.85	0.79
Phe + Tye,^c^%	1.36	1.32
Ca, %	0.83	0.75
Total P, %	0.66	0.60
Digestible P, %	0.43	0.36

^a^In each phase, two additional diets were formulated by adding *Bacillus subtilis* DSM 32540 or *Bacillus pumilus* DSM 32539 to the control diet, respectively. The dose for both probiotics was 500 mg/kg, which was equal to 1 × 10^9^ CFU/kg diet

^b^Provided the following quantities of vitamins and micro minerals per kilogram of complete diet: vitamin A as retinyl acetate, 11,136 IU; vitamin D_3_ as cholecalciferol, 2,208 IU; vitamin E as *DL*-alpha tocopheryl acetate, 66 IU; vitamin K as menadione dimethylprimidinol bisulfite, 1.42 mg; thiamin as thiamine mononitrate, 0.24 mg; riboflavin, 6.59 mg; pyridoxine as pyridoxine hydrochloride, 0.24 mg; vitamin B_12_, 0.03 mg; D-pantothenic acid as *D*-calcium pantothenate, 23.5 mg; niacin, 44.1 mg; folic acid, 1.59 mg; biotin, 0.44 mg; Cu, 20 mg as copper sulfate and copper chloride; Fe, 126 mg as ferrous sulfate; I, 1.26 mg as ethylenediamine dihydriodide; Mn, 60.2 mg as manganese sulfate; Se, 0.3 mg as sodium selenite and selenium yeast; and Zn, 125.1 mg as zinc sulfate

^c^Amino acids were indicated as standardized ileal digestible AA

After 7-d adaptation to the environment and diets, all pigs were orally inoculated with 3 mL per day of ETEC F18 for 3 consecutive days from d 0 post-infection (PI). The ETEC F18 expressing heat-labile, heat-stable and shiga-like toxins were originally isolated from a field disease outbreak by the University of Illinois Veterinary Diagnostic Lab (isolate number: U.IL-VDL # 05-27242). The inoculums were freshly prepared by the Western Institute for Food Safety and Security at UC Davis and were provided at 10^10^ CFU per 3 mL dose in phosphate buffer saline. This dose caused mild diarrhea in the current experiment, which is consistent with our previously published research [20, 21]. Briefly, ETEC F18 inoculum was prepared by propagating cell growth in Tryptic Soy broth (TSB, Difco/DB., USA) at 37 °C for 5 h with an orbital rotation of 150 r/min. After that, bacteria were harvested and separated from the supernatant of TSB broth through centrifugation at 10,000 r/min for 10 min. To achieve a homogenized inoculum with an approximate concentration of 3.3 × 10^9^ CFU/mL, bacterial cells were suspected in 1× phosphate buffer saline, then pooled, hand-shaken, and vortexed for 3 min. A total of 3 mL prepared inoculum (10^10^ CFU/syringe) was added to a sterile 5 mL syringe and stored on ice until used within 2 h of preparation.

### Clinical observations and sample collections

The procedures for conducting this experiment were adapted from the previously published research [17, 18]. Diarrhea scores were recorded daily from the first day of inoculation (d 0). The diarrhea score of each pig was assessed visually each day by two independent evaluators, with the score ranging from 1 to 5 (1 = normal feces, 2 = moist feces, 3 = mild diarrhea, 4 = severe diarrhea, and 5 = watery diarrhea). The frequency of diarrhea was calculated as the percentage of the pig days with diarrhea score 3 or greater, as well as calculated as the percentage of the pig days with diarrhea score 4 or greater.

Pigs and feeders were weighed at the beginning of the experiment, d 0 before inoculation, and d 7, d 14 and d 21 PI. Average daily gain (ADG), average daily feed intake (ADFI), and gain-to-feed ratio (G:F) was calculated for each interval from d − 7 to 0, d 0 to 7 PI, d 7 to 14 PI and d 14 to 21 PI. Fecal samples were collected from the rectum of all pigs on d 0 before inoculation, d 7, d 14 and d 21 PI using a fecal loop for the detection of β-hemolytic coliforms [20, 21]. Blood samples were collected from the jugular vein of all pigs with or without EDTA to yield whole blood and serum, respectively, before ETEC challenge (d 0 PI), and on d 3, 6, 13, and 21 PI. Whole blood samples (approximately 1 mL) were used for measuring total and differential blood cell count by the complete blood count. Serum samples were collected by centrifuging approximately 5 mL of whole blood samples at 20 °C at 1,500×*g* for 15 min. Serum samples were used to analyze for TNF-α and haptoglobin using porcine specific ELISA kits from R&D systems (Minneapolis, MN, USA) and Genway Biotech Inc. (San Diego, CA). Serum samples were diluted with dilution buffer at 1:10,000 prior to haptoglobin analysis. All samples were analyzed in duplicate and the procedures were similar to those described in our previously published research [20]. The intensity of the color was measured at 450 nm with the correction wavelength set at 530 nm. Concentrations were calculated from a standard curve.

All pigs were euthanized at the end of the experiment (d 21 PI). Before euthanization, pigs were anesthetized with 1 mL mixture of 100 mg telazol, 50 mg ketamine, and 50 mg xylazine (2:1:1) by intramuscular injection. After anesthesia, intracardiac injection with 78 mg sodium pentobarbital (Vortech Pharmaceuticals, Ltd., Dearborn, MI, USA) per 1 kg of BW was used to euthanize each pig. Jejunal and ileal mucosa samples were collected and immediately stored in liquid nitrogen for gene expression analysis. Four 3-cm segments from duodenum, the middle of the jejunum, ileum (10 cm close to the ileocecal junction), and distal colon were collected and fixed in Carnoy’s solution (ethanol, chloroform, and glacial acetic acid, 6:3:1 v/v/v) for intestinal morphology analysis. Mesenteric lymph nodes were aseptically collected and then pooled within pig, ground, diluted and plated on blood agar for measurement of total bacteria and the results were expressed as CFU per mg of lymph node [22, 23]. Spleen samples were analyzed in the same way as mesenteric lymph nodes for bacterial translocation. Digesta samples were collected from jejunum, ileum, and distal colon and immediately stored in liquid nitrogen for gut microbiome analysis.

### Detection of β-hemolytic coliforms

Briefly, fecal samples were plated in Columbia Blood Agar with 5% sheep blood to identify hemolytic coliforms, which can lyse red blood cells surrounding the colony. Fecal samples were also plated on MacConkey agar to enumerate total coliforms. Hemolytic colonies from the blood agar were sub-cultured on MacConkey agar to confirm that they were lactose-fermenting bacteria and flat pink colonies. All plates were incubated at 37 °C for 24 h in an air incubator. Populations of both total coliforms and β-hemolytic coliforms on blood agar were assessed visually, with a score from 0 to 8 (0 = no bacterial growth, 8 = very heavy bacterial growth). The ratio of scores of β-hemolytic coliforms to total coliforms was calculated. Questionable colonies were sub-sub-cultured on new MacConkey and blood agars to verify if they were β-hemolytic ETEC by using triple sugar iron agar and lysine iron agar and to verify if they were F18+ ETEC by means of a PCR [24].

### Intestinal morphology

The fixed intestinal tissues were embedded in paraffin, sectioned at 5 μm, and stained with high iron diamine and alcian blue. The slides were photographed by an Olympus BX51 microscope at 10× and all measurements were conducted in the image processing and analysis software (Image J, NIH). Fifteen straight and integrated villi and their associated crypts and surrounded area were selected to analyze villi height, crypt depth, the number of goblet cells per villus, and cross-sectional area of sulfo- and sialomucin as described by Kim et al. [21] and Deplancke and Gaskins [25].

### Quantitative real-time PCR

Jejunal and ileal mucosa samples were analyzed for gene expression by quantitative real-time PCR (qRT-PCR). Briefly, approximately 100 mg of mucosa sample was homogenized using TRIzol reagent (Invitrogen; Thermo Fisher Scientific, Inc., Waltham, MA). Then total RNA was extracted following RNA extraction procedural guidelines provided by reagent manufacturer. The RNA quality and quantity were assessed using an analyzer (Agilent 2100 Bioanalyzer; Agilent Technologies, Inc., Santa Clara, CA) and a spectrophotometer (Thermo Scientific, Inc., Waltham, MA). All samples used for further analysis had a ratio of optical density read at 260 and 280 nm around 2.0, a ratio of optical density read at 260 and 230 nm greater than 1.8. The cDNA was produced from 1 μg of total RNA per sample using the High-Capacity cDNA Reverse Transcription Kit (Applied Biosystems; Thermo Fisher Scientific, Inc., Waltham, MA) in a total volume of 20 μL. The mRNA expression of mucin 2 (*MUC2*), zona occludens-1 (*ZO-1*), claudin 1 (*CLDN1*), and occludin (*OCDN*) in jejunal mucosa and cyclooxygenase-2 (*PTGS2*), tumor necrosis factor alpha (*TNF*), Interleukin-1 beta (*IL1B*), and Interleukin 6 (*IL6*) in ileal mucosa were analyzed by qRT-PCR. Data normalization was accomplished using beta-actin (*ACTB*) and glyceraldehyde 3-phosphate dehydrogenase (*GAPDH*) as housekeeping genes. Primers were designed based on published literature and commercially synthesized by Integrated DNA Technologies, Coralville, IA [21]. All primers were verified prior to qRT-PCR (Supplementary Table 1). The qRT-PCR reaction conditions followed the published research [21, 26]. The 2^−ΔΔCT^ method was used to analyze relative quantification of genes compared with negative control [27].

### Gut microbiota in jejunum, ileum, and distal colon

Bacterial DNA was extracted from digesta samples using the Quick-DNA Fecal/Soil Microbe Kit (Zymo Research, Irvine, CA) following the manufacturer’s instructions. Extracted bacterial DNA was amplified with PCR, targeting the V4 region of the 16S rRNA gene with primers 515 F (5′- XXXXXXXXGTGTGCCAGCMGCCGCGGTAA-3′) with an 8 bp barcode (X) and Illumina adapter (GT) and 806 R (5′-GGACTACHVGGGTWTCTAAT-3′) [28]. Amplification included thermocycling conditions of 94 °C for 3 min for denaturation, 35 cycles of 94 °C for 45 s, 50 °C for 1 min, 72 °C for 1 .5 min, and 72 °C for 10 min (final elongation). To reduce polymerase chain reaction (PCR) bias, each sample was amplified in triplicate. Each PCR reaction included 2 μL of template DNA, 0.5 μL of barcode primer, 0.5 μL (10 μmol/L) of reverse primer, 12.5 μL of GoTaq 2X Green Master Mix (Promega, Madison, WI, USA), and 9.5 μL of nuclease free water. The triplicate PCR products were pooled and subjectively quantified based on the brightness of the bands on a 2% agarose gel with SYBR safe (Invitrogen Co., Carlsbad, CA, USA). All amplicons were then pooled at equal amounts. The pooled library was purified using the QIAquick PCR Purification Kit (Qiagen, Hilden, Germany) and submitted to the UC Davis Genome Center DNA Technologies Core for 250 bp paired-end sequencing on the Illumina MiSeq platform (Illumina, Inc. San Diego, CA, USA).

The software sabre (https://github.com/najoshi/sabre) was used to demultiplex and remove barcodes from raw sequences. Sequences were then imported into Quantitative Insights Into Microbial Ecology 2 (QIIME2; version 2018.6) for downstream filtering and bioinformatics analysis [29, 30]. Plugin q2-dada2 [31] was used for quality control and constructing features. Taxonomic classification was assigned using the feature-classifier plugin trained with SILVA rRNA database 99% Operational Taxonomic Units (OTU), version 132 [32, 33].

### Statistical analysis

Except for the microbiome data, normality of all other data were verified, and outliers were identified using the UNIVARIATE procedure (SAS Inst. Inc., Cary, NC, USA). Outliers were identified and removed as values that deviated from the treatment mean by more than 3 times the interquartile range. Data were analyzed by ANOVA using the PROC MIXED of SAS (SAS Institute Inc., Cary, NC, USA) in a randomized complete block design with the pig as the experimental unit. The statistical model included diet as the main effect and blocks (replicate and gender) as random effects. Treatment means were separated by using the LSMEANS statement and the PDIFF option of PROC MIXED. The Chi-square test was used for analyzing frequency of diarrhea. Statistical significance and tendency were considered at *P* < 0.05 and 0.05 ≤ *P* < 0.10, respectively.

Data visualization and statistical analysis for fecal microbiota were conducted using the R program (version 3.6.1). Two alpha diversity indices, Chao1 and Shannon, were calculated using the phyloseq package. Relative abundance was calculated using the phyloseq package and visualized using ggplot2 package in R. Relative abundance data were aggregated at various taxonomical levels. Shapiro-Wilk normality test and Bartlett test were used to verify normality and constant variance respectively in alpha diversity and relative abundance. Shannon index was analyzed using ANOVA with the statistical model including diets within different intestinal segment as fixed effects. Significance in Chao1 index and relative abundance was observed using Kruskal-Wallis rank sum test followed by a Conover test for multiple pairwise comparison using the agricolae package. Beta diversity was calculated based on the Bray-Curtis dissimilarity for principal coordinates analysis (PCoA). The homogeneity of multivariate dispersions was tested by the vegan package using the betadisper function, before the adonis function was used to calculate PERMANOVA with 999 replicate permutations.

## Results

### Growth performance and diarrhea score

All animals were healthy before ETEC challenge. A total of 8 pigs were removed from the whole data set due to health issues after ETEC infection or as outliers, including two pigs at the CON group, two pigs at the PRO1 group, and four pigs at the PRO2 group. No difference was observed in the BW of pigs among dietary treatments on d 0 before ETEC inoculation (Table 2). Compared with pigs in the CON group, ETEC challenged pigs supplemented with PRO1 tended to have greater (*P* < 0.10) BW on d 7 and 14 PI, greater (*P* < 0.10) ADG from d 0 to 7 PI and d 7 to 14 PI, and had significantly greater (*P* < 0.05) ADFI from d 7 to 14 PI. No differences were observed in any performance measurements between CON and PRO2 groups.

**Table 2:** Growth performance of enterotoxigenic *E. coli* F18 challenged pigs fed diets supplemented with probiotics

Item^c^	Control	PRO1^d^	PRO2^e^	SEM	*P*-value
BW, kg					
d − 7	7.56	7.48	7.78	0.397	0.20
d 0 PI	8.02	8.04	8.49	0.399	0.24
d 7 PI	9.48^b^	11.02^a^	10.28^ab^	0.420	0.069
d 14 PI	13.61^b^	16.30^a^	14.85^ab^	0.762	0.056
d 21 PI	18.86	21.13	19.51	1.077	0.31
ADG, g					
d − 7 to 0	86	77	103	19.39	0.63
d 0 to 7 PI	234^b^	416^a^	267^ab^	46.58	0.087
d 7 to 14 PI	583^b^	756^a^	644^ab^	49.18	0.091
d 14 to 21 PI	744	699	662	41.19	0.31
d 0 to 21 PI	517	624	523	50.19	0.24
Overall	409	486	419	39.58	0.30
ADFI, g					
d − 7 to 0	230	249	215	24.28	0.57
d 0 to 7 PI	493	589	552	69.03	0.33
d 7 to 14 PI	733^b^	1027^a^	877^ab^	70.62	< 0.05
d 14 to 21 PI	1016^ab^	1105^a^	910^b^	65.94	0.074
d 0 to 21 PI	745	907	742	64.95	0.14
Overall	616	742	611	52.61	0.15
Gain:Feed					
d − 7 to 0	0.366	0.377	0.477	0.068	0.18
d 0 to 7 PI	0.465	0.673	0.372	0.161	0.34
d 7 to 14 PI	0.802	0.743	0.699	0.042	0.27
d 14 to 21 PI	0.736^ab^	0.637^b^	0.742^a^	0.030	0.069
d 0 to 21 PI	0.695	0.689	0.689	0.026	0.98
Overall	0.662	0.656	0.670	0.020	0.90

^a,b^Means without a common superscript are different (*P* < 0.05)

^c^*BW* Body weight, *ADG* Average daily gain, *ADFI* Average daily feed intake, *PI* Post inoculation. Each least squares mean represents 8–10 observations

^d^PRO1 = *Bacillus subtilis* DSM 32540

^e^PRO2 = *Bacillus pumilus* DSM 32539

**Fig. 1:**
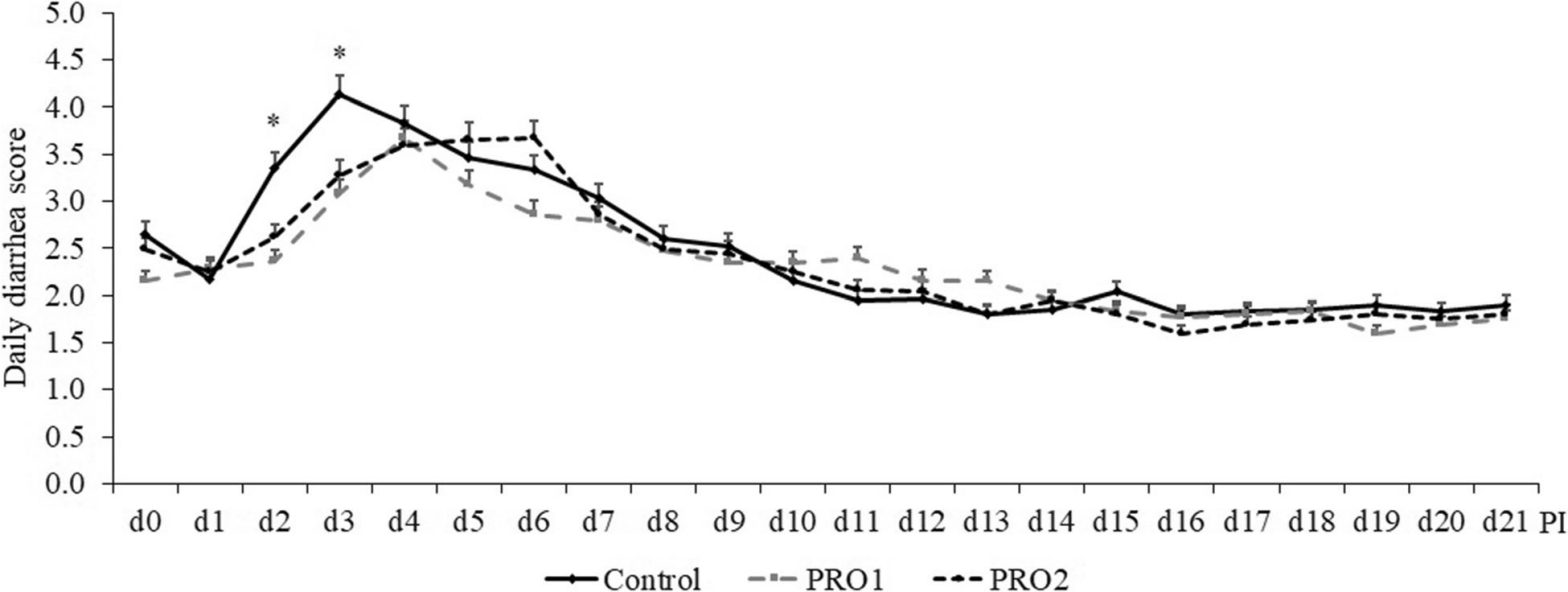
Daily diarrhea score of enterotoxigenic *E. coli* F18 challenged pigs fed diets supplemented with probiotics. Diarrhea score = 1, normal feces, 2, moist feces, 3, mild diarrhea, 4, severe diarrhea, 5, watery diarrhea. PI = post inoculation. *P < 0.05, indicating pigs in PRO1 group had lower diarrhea score than pigs in the control group on d 2 and 3 PI. Each least squares mean represents 8–10 observations. PRO1 = *Bacillus subtilis* DSM 32540; PRO2 = *Bacillus pumilus* DSM 32539

**Fig. 2:**
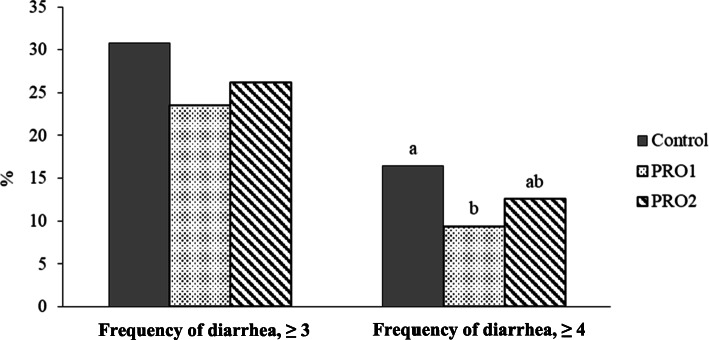
Frequency of diarrhea of enterotoxigenic *E. coli* F18 challenged pigs fed diets supplemented with probiotics. Frequency of diarrhea was calculated as the percentage of pig days with diarrhea score ≥ 3 or ≥ 4 in the total of pig days. ^a,b^Means without a common superscript are different (*P* < 0.05). Pigs in the PRO1 group had lower (*P* < 0.05) frequency of diarrhea throughout the experiment compared with pigs in the control group. Each least squares mean represents 8–10 observations. PRO1 = *Bacillus subtilis* DSM 32540; PRO2 = *Bacillus pumilus* DSM 32539

The daily diarrhea score peaked at d 2 to 6 PI after ETEC F18 infection (Fig. 1). Pigs supplemented with PRO1 had milder (*P* < 0.05) diarrhea on d 2 and d 3 PI and overall frequency of diarrhea than pigs in CON (Fig. 2). No difference was observed in diarrhea score and frequency of diarrhea between CON and PRO2, and between PRO1 and PRO2.

### Intestinal morphology

**Fig. 3:**
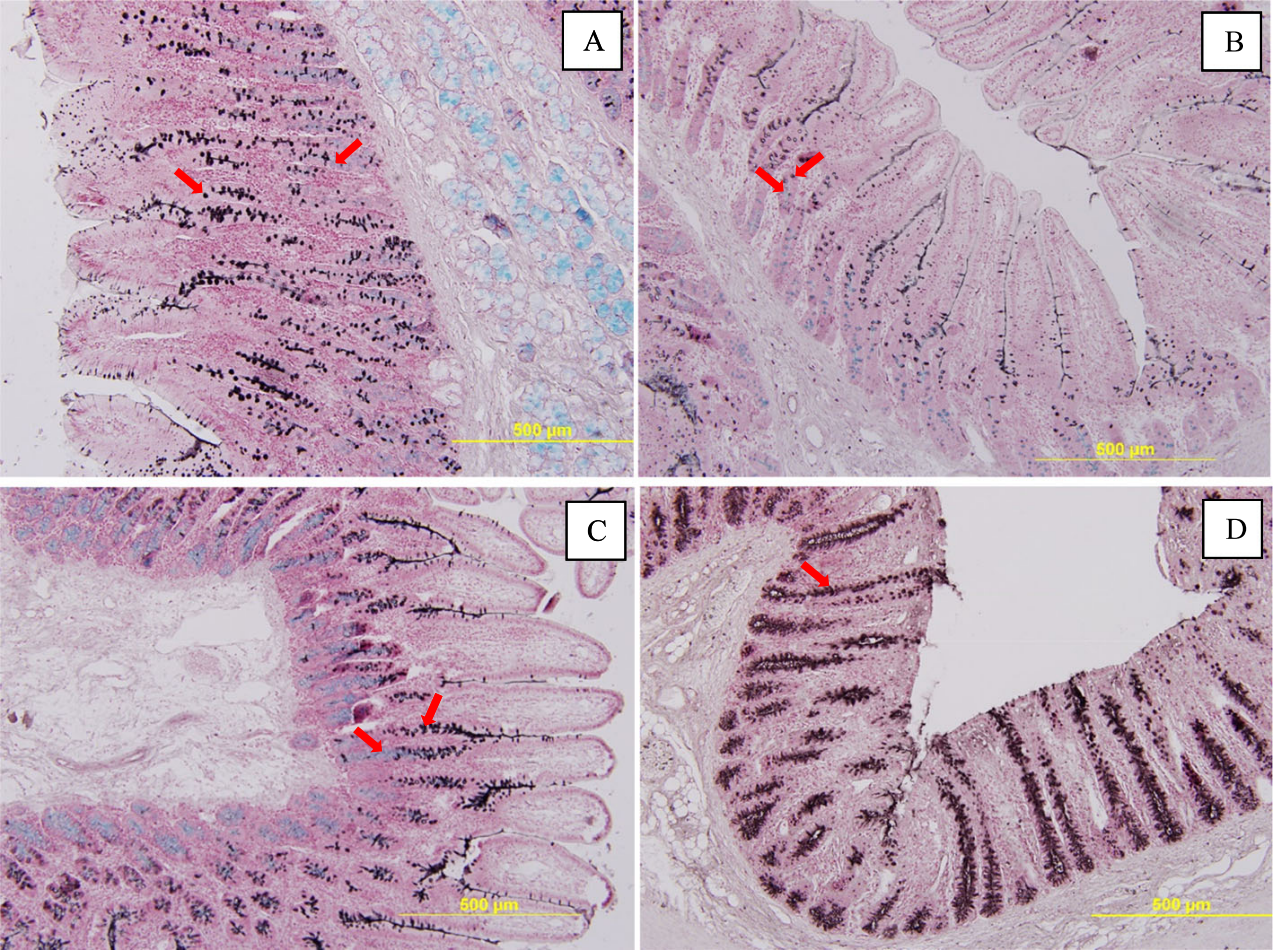
Sulfomucin (brown olor) and sialomucin (light blue color) in duodenum (**a**), jejunum (**b**), ileum (**c**), and colon (**d**) of weaned pigs

Sulfomucin and sialomucin were detected in duodenum, jejunum, and ileum, while only sulfamucin was shown in distal colon (Fig. 3). Sulfomucin was stained as brown color, whereas sialomucin was light blue color. Pigs supplemented with PRO1 had greater (*P* < 0.05) crypt depth and greater (*P* < 0.05) goblet cell number in duodenum and greater (*P* < 0.05) villi height in ileum compared with pigs in CON (Table 3). However, no difference was observed in sulfo- or sialomucin area in different segments of the intestine of pigs between CON and PRO1. Pigs supplemented with PRO2 had greater (*P* < 0.05) goblet cell number in duodenum, increased (*P* < 0.05) sulfomucin percentage in duodenal villi, and increased (*P* < 0.05) total mucin area and sialomucin percentage in jejunal villi, compared with CON. Compared to pigs in PRO2, pigs in PRO1 had increased (*P* < 0.05) duodenal crypt depth, increased (*P* < 0.05) Sialomucin area in the jejunum and increased (*P* < 0.05) crypt depth in the ileum.

**Table 3:** Intestinal morphology of enterotoxigenic *E. coli* F18 challenged pigs fed diets supplemented with probiotics

Item^c^	Control	PRO1^d^	PRO2^e^	SEM	*P*-value
Duodenum					
Villi height, μm	411	439	435	20.29	0.61
Crypt depth, μm	186^b^	224^a^	185^b^	9.19	< 0.05
Villi height: Crypt depth	2.21^ab^	1.98^b^	2.37^a^	0.091	< 0.05
Villi width, μm	187	200	185	5.81	0.10
Villi area, μm^2^	92,798	94,975	99,027	4718	0.50
Goblet cell number, per villi	24.88^b^	30.89^a^	32.31^a^	1.56	< 0.05
Total mucin area, % of villi area	6.79	7.50	9.42	1.05	0.30
Sulfomucin area, % of villi area	2.45^b^	3.21^ab^	4.80^a^	0.66	< 0.05
Sialomucin area, % of villi area	4.33	4.29	4.61	0.47	0.90
Jejunum					
Villi height, μm	479^a^	434^ab^	382^b^	17.25	< 0.01
Crypt depth, μm	169	158	151	7.73	0.33
Villi height: Crypt depth	2.89	2.79	2.53	0.15	0.28
Villi width, μm	130	128	127	5.58	0.88
Villi area, μm^2^	64,543^a^	59,882^ab^	54,277^b^	2795	0.08
Goblet cell number, per villi	15.11	15.57	16.40	0.71	0.52
Total mucin area, % of villi area	5.64^b^	7.72^ab^	9.39^a^	1.13	< 0.05
Sulfomucin area, % of villi area	3.06	4.07	3.43	0.79	0.61
Sialomucin area, % of villi area	2.60^b^	3.71^b^	5.88^a^	0.52	< 0.01
Ileum					
Villi height, μm	340^b^	386^a^	340^b^	14.43	< 0.05
Crypt depth, μm	161^ab^	182^a^	151^b^	8.76	0.07
Villi height: Crypt depth	2.14	2.17	2.27	0.12	0.71
Villi width, μm	137	149	130	7.14	0.28
Villi area, μm^2^	56,811	65,084	55,431	3829	0.27
Goblet cell number, per villi	19.33	17.31	17.57	2.09	0.77
Total mucin area, % of villi area	9.17	8.14	7.60	1.24	0.69
Sulfomucin area, % of villi area	5.81	5.24	5.05	0.99	0.87
Sialomucin area, % of villi area	3.36	2.85	2.68	0.40	0.48
Colon					
Crypt depth, μm	363	337	317	21.30	0.32
Sulfomucin area, % of villi area	40.96	42.40	44.85	2.67	0.65

^a,b^Means without a common superscript are different (*P* < 0.05)

^c^Each least squares mean represents 8–10 observations

^d^PRO1 = *Bacillus subtilis* DSM 32540

^e^PRO2 = *Bacillus pumilus* DSM 32539

### Complete blood counts and serum inflammatory markers

On day 0, no differences in white blood cells were observed among dietary treatments (Table 4). On d 3 PI, pigs in PRO1 had higher (*P* < 0.05) neutrophil percentages, lower (*P* < 0.05) lymphocytes percentages, and higher (*P* < 0.05) neutrophil to lymphocyte ratio compared with pigs in CON. Pigs in PRO2 had higher (*P* < 0.05) neutrophil count than pigs in CON. On d 6 PI, pigs in PRO1 had lower (*P* < 0.05) lymphocytes count, higher *(P* < 0.05) neutrophil percentage, lower (*P* < 0.05) lymphocyte percentage and higher (*P* < 0.05) neutrophil to lymphocyte ratio compared to pigs in CON. On d 13 PI, pigs in PRO1 had lower (*P* < 0.05) white blood cell count, lower (*P* < 0.05) lymphocyte count, lower (*P* < 0.05) lymphocyte percentages compared to pigs in CON. Pigs in PRO2 had lower (*P* < 0.05) lymphocyte percentages of white blood cell compared to pigs in CON. On d 21 PI, no differences in blood parameters were observed among treatments.

On d 0, no differences in inflammation markers were observed among dietary treatments (Table 4). On d 3 PI, pigs in PRO1 had lower (*P* < 0.05) concentration of haptoglobin compared to pigs in CON. On d 6 PI, pigs in both PRO1 and PRO2 had lower (*P* < 0.05) concentration of haptoglobin compared to pigs in CON. On d 13 PI, no differences in inflammation markers were observed between pigs in PRO1 and CON, but pigs in PRO2 had higher (*P* < 0.05) concentration of TNF-α compared to pigs in CON. On d 21 PI, no differences in inflammation markers were observed among dietary treatment.

On d 0, no differences in red blood cell profile were observed among dietary treatments. On d 3 PI, pigs in PRO2 had greater (*P* < 0.05) red cell distribution width, greater (*P* < 0.05) total protein compared to pigs in CON (Supplementary Table 2). Supplementation of PRO1 had increased (*P* < 0.05) mean corpuscular hemoglobin concentration and reduced (*P* < 0.05) total protein compared with PRO2. On d 6 PI, pigs in PRO1 had lower (*P* < 0.05) hemoglobin concentration, lower (*P* < 0.05) packed cell volume and lower (*P* < 0.05) total protein compared to pigs in CON. Pigs in PRO2 had greater (*P* < 0.05) red cell distribution width compared to pigs in CON. Supplementation of PRO1 reduced (*P* < 0.05) packed cell volume and total protein compared with PRO2. On d 13 PI, pigs in PRO1 had lower (*P* < 0.05) hemoglobin concentration, lower (*P* < 0.05) packed cell volume and lower (*P* < 0.05) platelets counts compared to pigs in CON. Pigs in PRO2 had greater (*P* < 0.05) total protein compared with pigs in CON. On d 21 PI, pigs in PRO1 had greater (*P* < 0.05) mean platelet volume compared to pigs in PRO2.

**Table 4:** Total and differential white blood cells and serum inflammatory markers in enterotoxigenic *E. coli* F18 challenged pigs fed diets supplemented with probiotics

Item^c^	Control	PRO1^d^	PRO2^e^	SEM	*P*-value
D 0 before inoculation					
WBC, 10^3^/μL	13.17	13.20	13.67	1.10	0.94
Neu, 10^3^/μL	6.24	6.72	6.80	0.64	0.69
Lym, 10^3^/μL	5.77	5.40	6.11	0.50	0.67
Mono, 10^3^/μL	1.03	0.84	0.83	0.17	0.67
Eos, 10^3^/μL	0.064	0.094	0.076	0.025	0.59
Baso, 10^3^/μL	0.022	0.019	0.034	0.009	0.48
Neu, % of WBC	47.74	51.39	49.31	2.34	0.41
Lym, % of WBC	44.07	42.05	44.24	2.12	0.62
Mono, % of WBC	7.51	5.75	6.22	0.80	0.33
Eos, % of WBC	0.49	0.58	0.58	0.14	0.84
Baso, % of WBC	0.15	0.13	0.24	0.056	0.38
Neu:Lym	1.12	1.27	1.20	0.12	0.56
TNF-α, pg/mL	75.73	69	66.36	23.9	0.96
Haptoglobin, μg/mL	1080	1282	1185	135.41	0.58
D 3 post inoculation					
WBC, 10^3^/μL	17.34	18.09	20.35	0.97	0.10
Neu, 10^3^/μL	8.82^b^	10.64^ab^	11.33^a^	0.71	0.069
Lym, 10^3^/μL	7.46	6.34	7.74	0.59	0.30
Mono, 10^3^/μL	0.91	0.97	1.12	0.14	0.62
Eos, 10^3^/μL	0.11	0.11	0.12	0.031	0.96
Baso, 10^3^/μL	0.047	0.031	0.040	0.011	0.63
Neu, % of WBC	51.03^b^	59.81^a^	54.73^ab^	2.47	< 0.05
Lym, % of WBC	42.68^a^	34.67^b^	39.04^ab^	2.28	< 0.05
Mono, % of WBC	5.45	5.35	5.50	0.74	0.99
Eos, % of WBC	0.59	0.55	0.573	0.15	0.95
Baso, % of WBC	0.25	0.15	0.19	0.049	0.41
Neu:Lym	1.23^b^	1.81^a^	1.56^ab^	0.18	< 0.05
TNF-α, pg/mL	59.03	24.9	87.18	32.15	0.42
Haptoglobin, μg/mL	2005^a^	1320^b^	1444^ab^	277.1	< 0.05
D 6 post inoculation					
WBC, 10^3^/μL	23.85^a^	17.59^b^	20.29^ab^	1.66	< 0.05
Neu, 10^3^/μL	10.94	9.66	10.02	1.213	0.73
Lym, 10^3^/μL	11.66^a^	7.13^b^	9.06^ab^	0.89	< 0.05
Mono, 10^3^/μL	1.05	0.72	0.94	0.12	0.19
Eos, 10^3^/μL	0.15^a^	0.06^b^	0.19^a^	0.027	< 0.05
Baso, 10^3^/μL	0.061	0.021	0.054	0.014	0.17
Neu, % of WBC	45.56^b^	55.41^a^	48.76^ab^	2.83	< 0.05
Lym, % of WBC	49.10^a^	40.13^b^	46.85^ab^	2.86	< 0.05
Mono, % of WBC	4.56	3.93	4.37	0.49	0.57
Eos, % of WBC	0.59^ab^	0.33^b^	0.92^a^	0.11	< 0.05
Baso, % of WBC	0.25	0.13	0.27	0.063	0.32
Neu:Lym	0.98^b^	1.45^a^	1.18^ab^	0.14	< 0.05
TNF-α, pg/mL	75.4	30.11	68.83	39.61	0.55
Haptoglobin, μg/mL	1652^a^	914^b^	906^b^	206.37	< 0.05
D 13 post inoculation					
WBC, 10^3^/μL	16.62^a^	12.73^b^	15.50^ab^	1.40	0.087
Neu, 10^3^/μL	7.15	6.12	8.03	0.81	0.34
Lym, 10^3^/μL	8.60^a^	5.90^b^	6.87^ab^	0.65	< 0.05
Mono, 10^3^/μL	0.72	0.61	0.70	0.079	0.52
Eos, 10^3^/μL	0.077^ab^	0.040^b^	0.138^a^	0.022	< 0.05
Baso, 10^3^/μL	0.019	0.020	0.021	0.005	0.93
Neu, % of WBC	42.86	48.35	48.53	1.90	0.12
Lym, % of WBC	52.03^a^	46.27^b^	45.53^b^	1.65	< 0.05
Mono, % of WBC	4.54	4.98	4.84	0.53	0.85
Eos, % of WBC	0.45^ab^	0.29^b^	0.97^a^	0.189	< 0.05
Baso, % of WBC	0.11	0.16	0.13	0.059	0.21
Neu:Lym	0.84^b^	1.06^ab^	1.11^a^	0.074	< 0.05
TNF-α, pg/mL	21.83^b^	21.19^b^	67.44^a^	13.03	0.08
Haptoglobin, μg/mL	847.6	460.2	894.9	142.35	0.09
D 21 post inoculation					
WBC, 10^3^/μL	15.38	14.97	14.51	1.43	0.93
Neu, 10^3^/μL	7.05	6.80	6.65	0.73	0.94
Lym, 10^3^/μL	6.87	6.84	6.56	0.74	0.95
Mono, 10^3^/μL	1.17	1.12	1.08	0.18	0.95
Eos, 10^3^/μL	0.23	0.21	0.21	0.056	0.95
Baso, 10^3^/μL	0.038	0.030	0.044	0.012	0.69
Neu, % of WBC	45.28	45.74	45.77	2.38	0.99
Lym, % of WBC	45.60	45.31	45.15	2.76	0.99
Mono, % of WBC	7.41	7.13	7.48	0.78	0.95
Eos, % of WBC	1.45	1.59	1.37	0.35	0.91
Baso, % of WBC	0.24	0.21	0.24	0.068	0.93
Neu:Lym	1.03	1.04	1.16	0.13	0.76
TNF-α, pg/mL	69.46	33.46	143.31	37.67	0.19
Haptoglobin, μg/mL	272	267	241	84.96	0.96

^a,b^Means without a common superscript are different (*P* < 0.05)

^c^*WBC* White blood cell, *Neu* Neutrophil, *Lym* Lymphocyte, *Mono* Monocyte, *Eos* Eosinophil, *Baso* Basophil. Each least squares mean represents 8–10 observations

^d^PRO1 = *Bacillus subtilis* DSM 32540

^e^PRO2 = *Bacillus pumilus* DSM 32539

### Gene expression

**Fig. 4:**
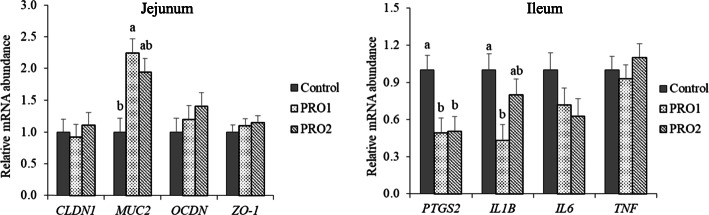
Relative mRNA expression of *CLDN1*, *MUC2*, *OCDN*, and *ZO-1* in jejunal mucosa and relative mRNA expression of *PTGS2, IL1B, IL6,* and *TNF* in ileal mucosa of enterotoxigenic *E. coli* F18 challenged pigs fed diets supplemented with probiotics on d 21 post inoculation. ^a,b^Means without a common superscript are different (*P* < 0.05). Each least squares mean represents 8–10 observations. PRO1 = *Bacillus subtilis* DSM 32540; PRO2 = *Bacillus pumilus* DSM 32539

Pigs supplemented with PRO1 had increased (*P* < 0.05) mRNA expression of *MUC2* in jejunal mucosa and decreased (*P* < 0.05) mRNA expression of *PTGS2* and *IL1B* in ileal mucosa, compared with pigs in the CON group (Fig. 4). Pigs fed with PRO2 had decreased (*P* < 0.05) mRNA expression of *PTGS2* in ileal mucosa, compared with pigs in the CON group. No differences were observed between in the expression of tested genes between PRO1 and PRO2 groups.

### Bacterial translocation

**Fig. 5:**
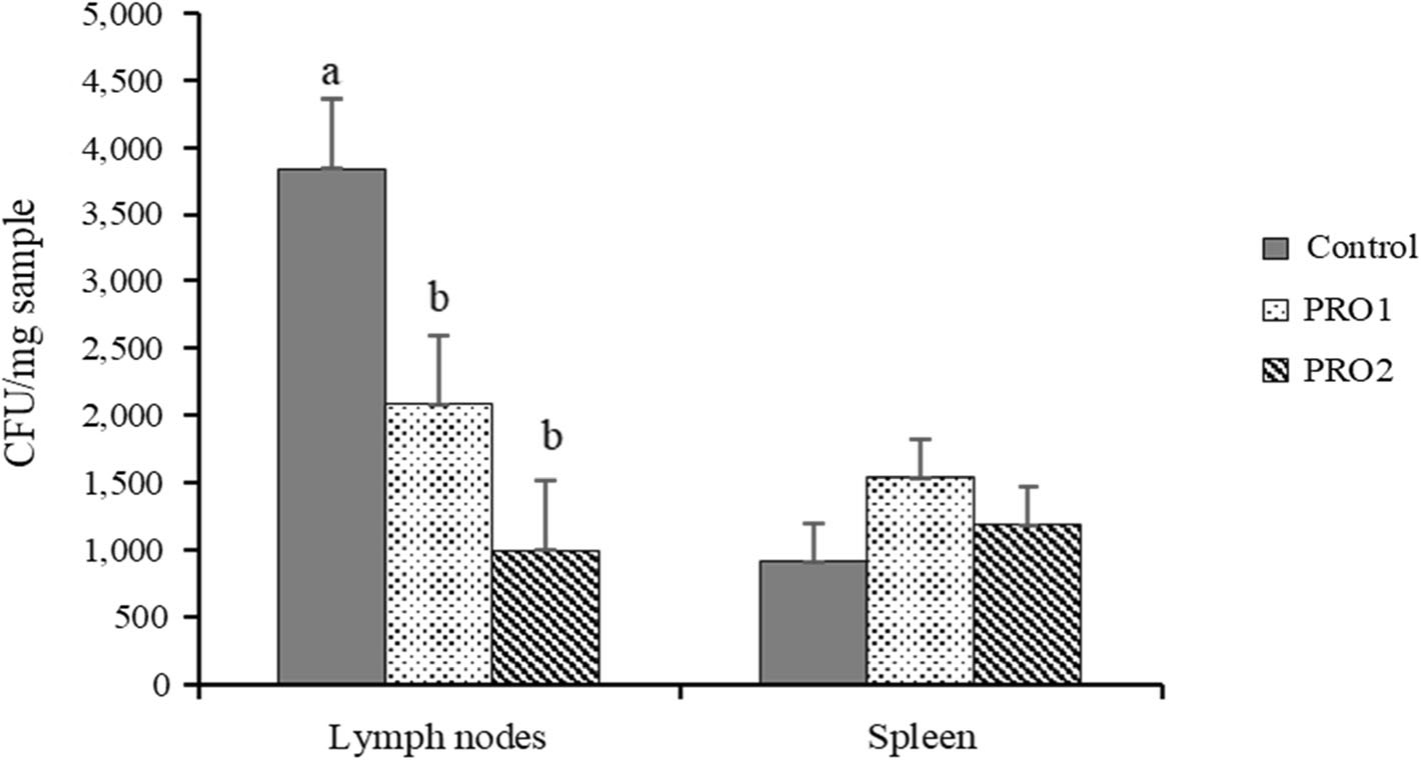
Total coliforms in mesenteric lymph nodes and spleen of enterotoxigenic *E. coli* F18 challenged pigs fed diets supplemented with probiotics on d 21 post inoculation. ^a,b^Means without a common superscript are different (*P* < 0.05). Pigs supplemented with probiotics PRO1 or PRO2 had less (*P* < 0.05) total coliforms in mesenteric lymph nodes compared with pigs in the control group. Each least squares mean represents 8–10 observations. PRO1 = *Bacillus subtilis* DSM 32540; PRO2 = *Bacillus pumilus* DSM 32539

Pigs in PRO1 and PRO2 had reduced (*P* < 0.05) total coliforms in mesenteric lymph nodes (2083 and 996 CFU/mg sample, Fig. 5) compared with pigs in CON (3838 CFU/mg sample) on d 21 PI. No difference was observed in total coliforms in spleen among three dietary treatments.

### Gut microbiota

**Fig. 6:**
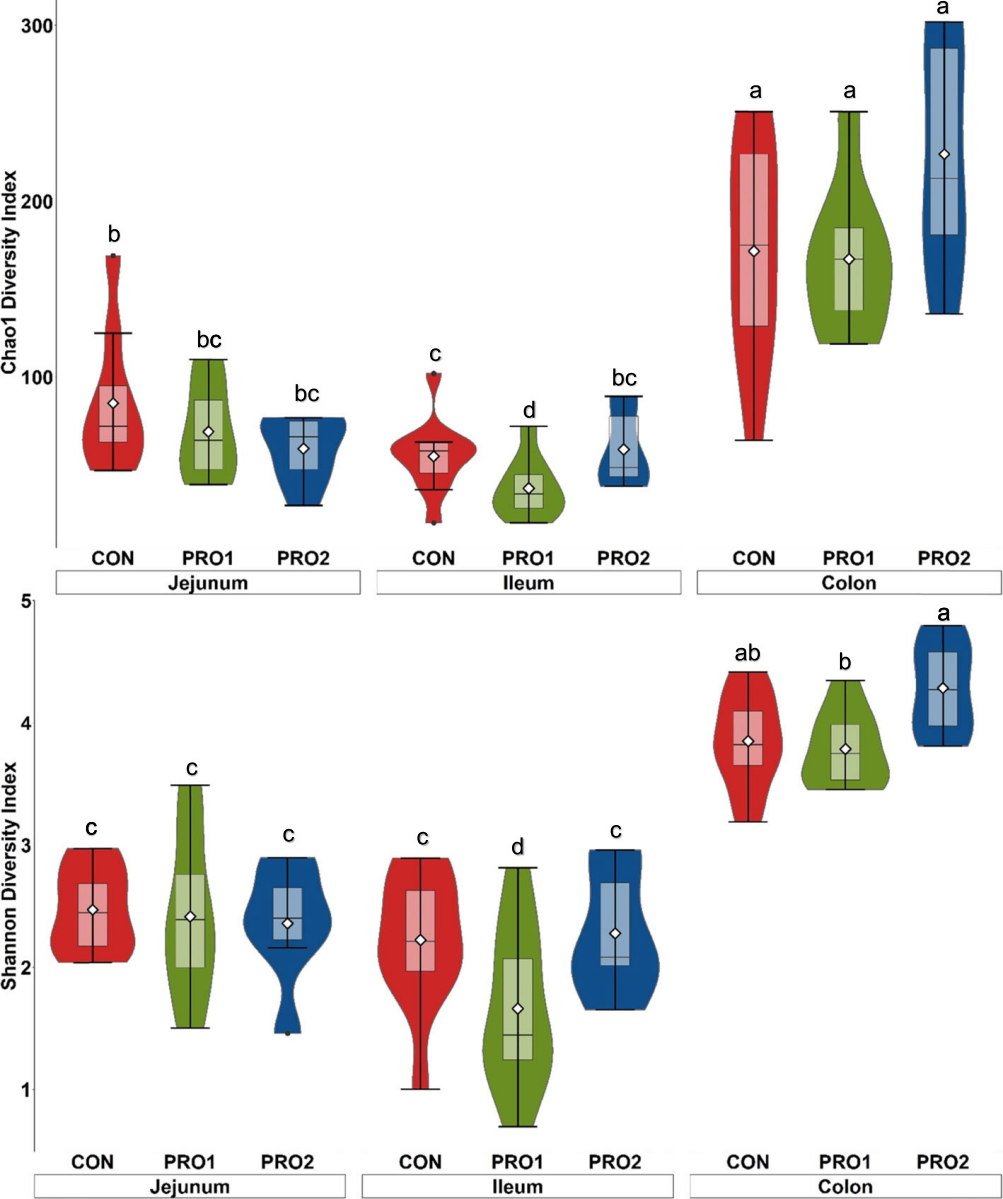
Alpha diversity as indicated by Chao1 (A) and Shannon (B) in intestinal content collected at jejunum, ileum, and distal colon of enterotoxigenic *E. coli* F18 challenged pigs fed diets supplemented with probiotics on d 21 post inoculation. ^a-d^Means without a common superscript are different (*P* < 0.05). Each least squares mean represents 7–10 observations. PRO1 = *Bacillus subtilis* DSM 32540; PRO2 = *Bacillus pumilus* DSM 32539

**Fig. 7:**
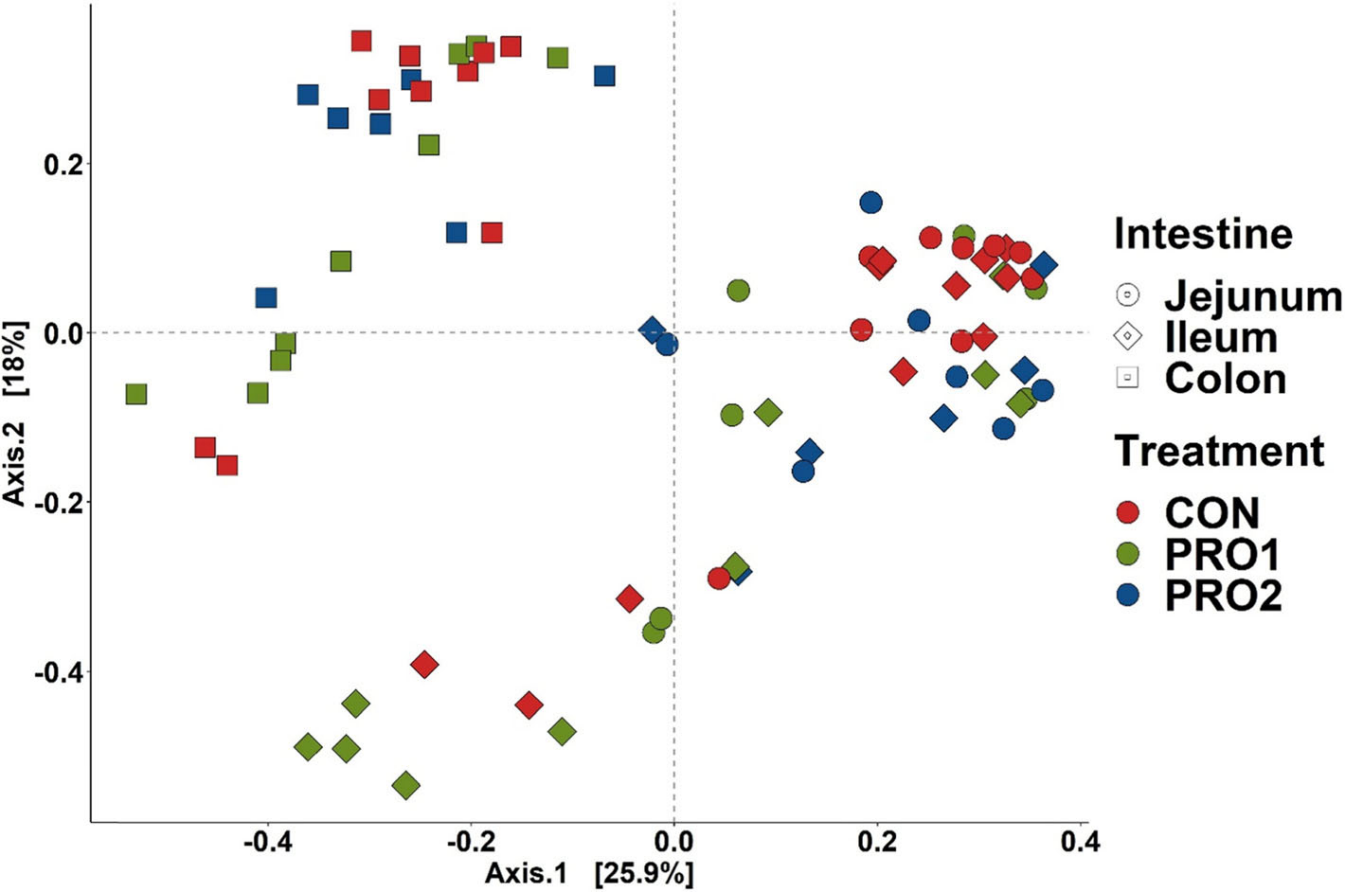
Beta diversity of intestinal microbiota in the jejunum, ileum, and distal colon of enterotoxigenic *E. coli* F18 challenged pigs fed diets supplemented with probiotics on d 21 post inoculation. Data were analyzed by principal coordinate analysis (PCoA) based on the Bray-Curtis dissimilarity. Symbols indicate dietary treatments and colors indicate different dates. PRO1 = *Bacillus subtilis* DSM 32540; PRO2 = *Bacillus pumilus* DSM 32539

A total of 1,327,261 qualified reads were obtained with a mean of 17,066 reads per sample. A total of 7,796 OTUs were identified in the current experiment. Following the order of the intestinal segments (jejunum, ileum, and colon), the average OTUs were 85, 55, and 172 in CON, the average OTUs were 69, 37, 167 in PRO1, whereas the average OTUs were 59, 59, and 227 in PRO2. Both Chao1 and Shannon indices in the colonic content were significantly higher (*P* < 0.05) than that in jejunal and ileal content, regardless of dietary treatments (Fig. 6). Pigs supplemented with PRO1 had lower (*P* < 0.05) Chao1 and Shannon indices in ileal content than pigs in CON and PRO2. No difference was observed in the alpha diversity of jejunal and distal colon content among dietary treatments. For beta diversity (Bray-Curtis distance), compositional differences of the intestinal microbiota were observed among jejunum, ileum, and distal colon (Adonis, *P* < 0.05; Fig. 7). Compositional differences of the intestinal microbiota at phyla level were also observed between PRO1 and CON groups (Pairwise-Adonis, *P* < 0.05; Fig. 7).

**Fig. 8:**
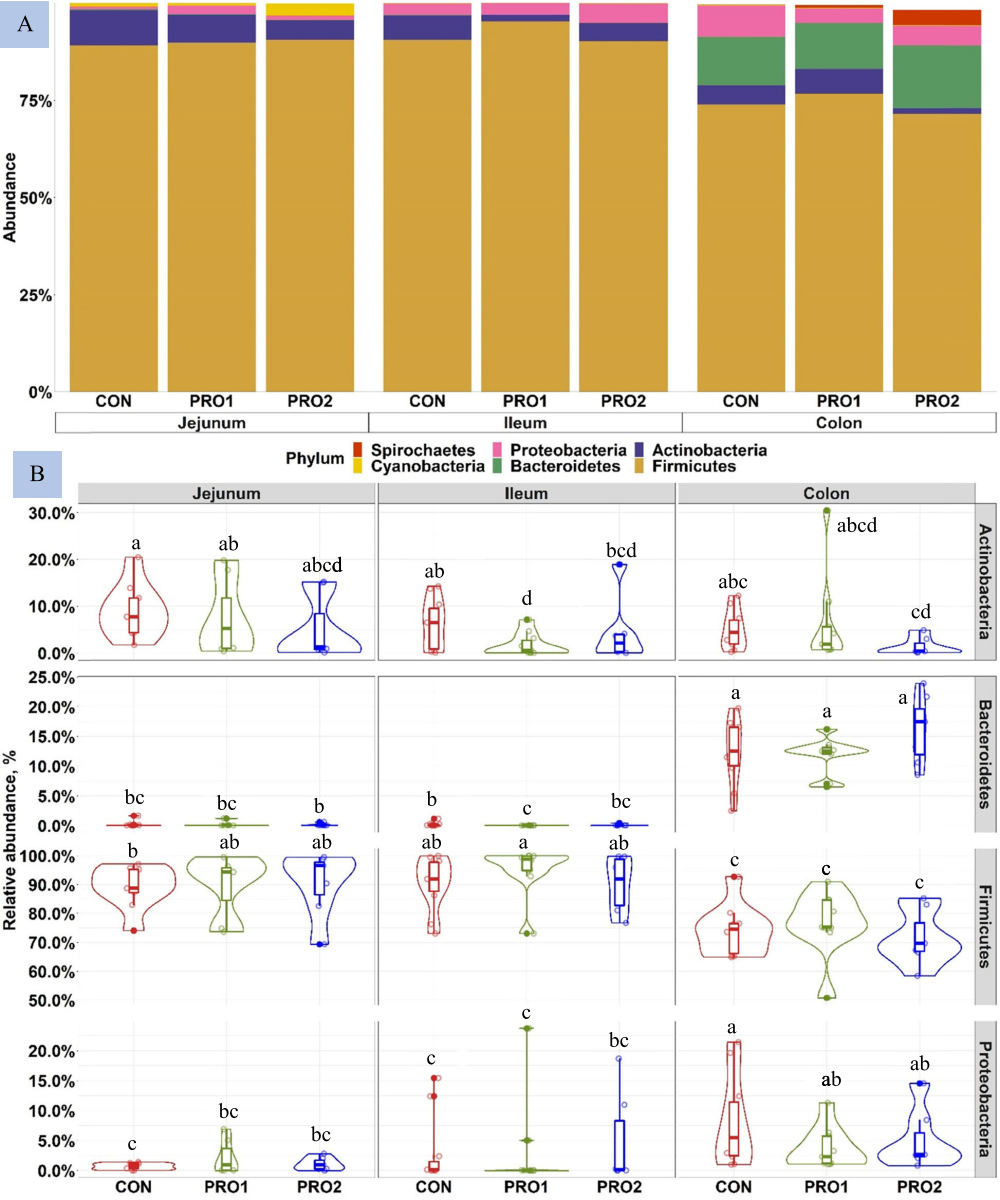
Stacked bar plot showing the relative abundance of bacterial phyla in the jejunum, ileum, and distal colon of enterotoxigenic *E. coli* F18 challenged pigs fed diets supplemented with probiotics on d 21 post inoculation (**A**). Violin plot showing the relative abundance of individual bacterial phylum (**B**). ^a-c^Means without a common superscript are different (*P* < 0.05). Each least squares mean represents 7–10 observations. PRO1 = *Bacillus subtilis* DSM 32540; PRO2 = *Bacillus pumilus* DSM 3253

On d 21 PI, the top 3 phyla in the jejunal and ileum content were Firmicutes, Actinobacteria, and Proteobacteria (Fig. 8), whereas the distal colonic content was dominated by Firmicutes, Bacteroidetes, Actinobacteria, and Proteobacteria. Pigs in the PRO1 group had lower (*P* < 0.05) relative abundance of Actinobacteria and Bacteroidetes in the ileum than pigs in CON. Within Firmicutes phylum, pigs supplemented with PRO1 had lower (*P* < 0.05) relative abundance of Erysipelotrichaceae (0.40% vs. 2.50%), Lachnospiraceae (0.11% vs. 0.36%), and Peptostreptococcaceae (0.59% vs. 2.89%) in ileal content than pigs in CON (Supplementary Table 3; Supplementary Fig. 1). Pigs supplemented with PRO2 had lower (*P* < 0.05) the relative abundance of Lachnospiraceae (0.40% vs. 1.31%) and Ruminococcaceae (0.12% vs. 0.44%) in jejunal content, compared with pigs in CON. Within Actinobacteria and Proteobacteria phyla, supplementation of PRO1 reduced (*P* < 0.05) the relative abundance of Atopobiaceae (0.05% vs. 0.37%) and Bifidobacteriaceae (1.67% vs. 5.86%), but increased the relative abundance of Pasteurellaceae (2.80% vs. 2.62%) in ileal content, compared with pigs in CON. Supplementation of PRO2 decreased (*P* < 0.05) the relative abundance of Atopobiaceae (0.18% vs. 1.28%), Bifidobacteriaceae (3.55% vs. 7.37%), and Pasteurellaceae (0.02% vs. 0.21%) in jejunal content, compared with pigs in CON.

## Discussion

Results from the present study demonstrated that dietary supplementation of *Bacillus subtilis* DSM 32540 reduced severity of diarrhea, enhanced growth performance, alleviated systemic inflammation, and modified gut health of ETEC F18 challenged pigs. However, the reference strain *Bacillus pumilus* DSM 32539 had limited effects on performance and health of weaned pigs in comparison to DSM 32540, which was also supported by the pig removal during the experiment. The beneficial effects of *Bacillus subtilis* DSM 32540 on growth performance and disease resistance may be attributed to several mechanisms including but not limited to: 1) reduction of pathogenic ETEC population in the gastrointestinal tract through production of antimicrobials (not tested in the current research), 2) enhancement of the host gut health and modification of gut microbiota, and 3) regulation of host immune system [34–36]. The last two potential mechanisms are discussed below based on the analysis in the current experiment.

Post-weaning diarrhea caused by ETEC is of great economic importance in the swine industry. The disease was shown to reduce growth performance and increase mortality rate of weaning pigs during the first 2 weeks after weaning [37]. A study by Amezcua et al. [38] reported that on overage, nursery pigs in farms having *E. coli* problem had a decrease of ADG from 452 g/d to 414 g/d and an increase of mortality from 2% to 7%. The present results have shown that supplementation of probiotics *Bacillus subtilis* DSM 32540 had remarkably reduced severity of diarrhea, which was consistent with the improved growth performance of these pigs after ETEC F18 challenge. Findings of this experiment are also consistent with previously published research that claimed the positive effects of *Bacillus subtilis* on performance and diarrhea of weaned pigs under healthy [12, 15] and disease challenge conditions [21, 39, 40].

A heathy gut is critically important for disease resistance and growth of weaned pigs. Previous research has reported that ETEC induced post-weaning diarrhea is highly correlated with disrupted intestinal structure and functions as a digestive and absorptive organ and a physical barrier [41, 42]. The toxins secreted by ETEC could induce loss of intestinal villous cells and the consequent villus atrophy, which further decrease the digestive and absorptive capacity of pigs and cause reduced performance [43, 44]. In addition, post-weaning diarrhea is also associated with increased gut permeability due to the disturbed tight junction protein expression in the small intestine [34]. In the current study, supplementation of *Bacillus subtilis* DSM 32540 increased ileal villi height and duodenal crypt depth compared with pigs in the control group on d 21 PI, which was also reported in other studies where the same *Bacillus subtilis* strain was used in pigs and chickens under normal housing conditions [45, 46]. The villus is populated by differentiated enterocytes that have distinctive absorptive and secretary functions, while the crypt is populated by stem cells that are rapidly proliferating and differentiating [47]. Generally, a decrease in villus height to crypt depth ratio may indicate a reduced absorptive and secretary capacity. This is likely due to an accelerated crypt cell turnover rate which may result in insufficient time for migrating cells to be fully differentiated [48]. In the present study, the increase in crypt depth may be a source of energy expenditure [49]. However, the energy spent may be compensated by the effect of increased ileal villus height as reflected in the enhanced growth performance of pigs. The exact mechanism by which *Bacillus subtilis* DSM 32540 promotes cell proliferation may need further investigation. Nonetheless, previously published research has indicated that the stimulation of crypt cell proliferation may be mediated by short chain fatty acids produced from gram-positive bacteria [50]. Additionally, *Bacillus subtilis* DSM 32540 was shown to be able to promote epithelial cell proliferation by upregulating the mRNA expression of epidermal growth factor and glucagon-like peptide 2 in the ileum [46]. However, supplementation of probiotics did not impact the mRNA expression of claudin, occludin and zona occludens-1 in jejunal mucosa on d 21 PI. These observations are consistent with the results of daily diarrhea score confirmed that pigs have fully recovered from the deleterious effects of ETEC F18 infection after 3 weeks.

Another consequence of a perturbed gut integrity and permeability is increased bacterial translocation from intestinal lumen into the distant organs [51]. Bacterial translocation describes the passage of indigenous or non-indigenous bacteria from gastrointestinal tract to other external organs [52]. Bacterial translocation happens naturally and continuously in the gastrointestinal tract of an animal. However, it may be affected by the change of gastrointestinal microbiota and the damaged gut integrity due to abnormal exposure to pathogens or toxins [53]. In general, mesenteric lymph nodes and other organs are sterile in a healthy and immunocompetent animal because this small population of indigenous bacteria are killed either during the passage or in the lymph organs by the reticuloendothelial system [54]. In the present study, pigs supplemented with both probiotic *Bacillus* spp*.* strains had lower bacterial populations in the mesenteric lymph nodes than pigs in the control group. Although the increase in mRNA expression of tight junction proteins was not observed in the jejunum of weaned pigs from both *Bacillus* spp*.* groups, the reduced bacterial translocation suggests that pigs in the probiotic groups may have improved gut integrity and barrier function. Future research may take this into consideration.

The secreted mucus (i.e., glycoprotein mucin 2) from goblet cells forms gel-matrix that retains antibacterial proteins such as RegIIIγ, and physically separate pathogens with underlying intestinal epithelial cells [55, 56]. The upregulation of *MUC2* expression by probiotic supplementation suggests the regulatory effects of both *Bacillus* strains on mucin production in the small intestine. The lipoteichoic acid structure and the metabolites from *Bacillus* strains may attribute to the regulation of *Bacillus* on mucin production [57, 58]. However, limited impacts were observed in the mucin types (sulfo- and sialo-mucin) in the intestine of pigs when they were supplemented with probiotics. Sulfomucin and sialomucin are acidic mucins secreted by goblet cells in the gastrointestinal tract. Newly formed goblet cells migrate up the crypt and villi, switching the production of sialomucin to sulfomucin as they mature [59]. It was shown that sulfomucin is more resistant against bacterial enzymatic degradation due to high levels of sulphate in the mucin [60]. This was also the reason that sulfomucin was dominant in the distal colon, instead of sialomucin. Pigs supplemented with *Bacillus pumilus* DSM 32539 had more goblet cells and sulfomucin area in duodenum. However, this was not the case in jejunal villi. Overall, these observations indicate that *Bacillus pumilus* DSM 32539 may have stronger impacts on goblet cells in the small intestine than *Bacillus subtilis* DSM 32540, which needs further investigation in the future research, especially as the control strain did not exert the phenotypic effects on the piglets with improved growth performance or reduced diarrhea that the probiotic strain did.

Our previously published research demonstrated that oral inoculation of ETEC F18 could induce systemic inflammation of weaned pigs by increasing total white blood cell counts, neutrophils, and lymphocytes with the peak of inflammation at approximately d 5 to 6 PI [20]. In the present study, pigs supplemented with *Bacillus subtilis* DSM 32540 had lower total white blood cell counts and lymphocytes at the peak of ETEC infection than pigs in the control group. Consistently, an acute phase protein, haptoglobin was also lower in the serum samples collected from pigs supplemented with *Bacillus subtilis* DSM 32540. These observations indicate pigs in this probiotic group had less severe systemic inflammation compared with pigs in the control group [20, 61]. Similar results were also observed in intestinal inflammation, as indicated by the mRNA expression of *PTGS2* and *IL1B* in ileal mucosa were downregulated by *Bacillus subtilis* DSM 32540 supplementation. *PTGS2* encodes cyclooxygenase-2, the inducible form of prostaglandin synthetase, which catalyzes the committed step in the prostaglandin production pathway [62]. Our previous research reported that ETEC F18 challenge remarkably upregulated *PTGS2* expression in ileal mucosa of weaned pigs [26]. The reduced expression of *PTGS2* indicates that pigs in *Bacillus subtilis* DSM 32540 group had reduced gut inflammation compared with pigs in the control group. The mRNA expression of pro-inflammatory cytokines, including *IL1B, IL6* and *TNF*, were also analyzed in the ileal mucosa of weaned pigs. Supplementation of *Bacillus subtilis* DSM 32540 reduced *IL1B* expression compared with pigs in the control, but no differences were observed in *IL6* and *TNF* among dietary treatments. This was not surprising because the responses of inflammatory cytokines may be different due to the age of animal and the severity of bacterial infection. It has been reported that IL-1β could induce cell apoptosis locally and exert anorexic effect such as feed intake suppression [63]. Therefore, a decrease in mRNA expression of *PTGS2* and *IL1B* is beneficial for pigs in terms of their feed intake and growth performance.

The composition and diversity of gut microbiota in pigs is highly impacted by their healthy status and nutrient components that are offered in animal feed [64, 65]. One of the potential modes of action for using probiotics in feed to improve overall gut health is the increase of favorable bacteria population in the gut or maintenance of a favorable balance in the gut ecosystem. To test the impacts of probiotic supplementation on the gut microbiota diversity along the intestinal tract, intestinal contents were collected from middle of jejunum, ileum, and distal colon and 16S rRNA sequencing was performed. In consistent with previously published research, the microbial diversity between jejunum and ileum was similar, and distal colon had the highest microbial diversity [66, 67]. However, supplementation of either *Bacillus* spp. did not impact microbiome diversity in the contents of jejunum, ileum, and colon. Alpha rarefaction curve (Supplementary Fig. 2) in the present study showed that a reasonable number of reads have been determined by 16S rRNA sequencing. The bacterial richness estimation ranged from 37 to 227 OTUs/sample, which, however, were lower than previously published research using the same procedures [66, 68]. It is important to note that many clustering methods, such as OTU clustering algorithms, diminishing sequencing errors, during microbiome analysis may attribute to these differences at taxonomic level [69].

Firmicutes was the most dominant phylum in all segments and was followed by Bacteroidetes in the colon [65, 70]. The relatively high abundance of Firmicutes and Bacteroidetes in the intestine is likely due to the plant-based ingredients that were used in the diets [71]. Both phyla benefit the host by being actively involved in host’s carbohydrate metabolism, amino acid metabolism, and short-chain fatty acids production, thus play important roles in energy production and maintenance of a healthy gut [72, 73].

The results in the current study indicate that *Bacillus subtilis* DSM 32540 impacts more on ileal microbiota, while *Bacillus pumilus* DSM 32539 has more effects on jejunal microbiota. The spore-formed *Bacillus* spp. are highly resilient and retain their viability during storage. However, they need to regain their metabolic activity after ingested by the host. Therefore, understanding their lifecycle, including their germination and colonization in the different intestinal segments, will be of interest to decipher their different impacts [74]. Interestingly, although the impacts of tested *Bacillus* spp. were different in location, both *Bacillus* spp. reduced similar bacterial families including Lachnospiraceae, Atopobiaceae*,* Bifidobacteriaceae, and Pasteurellaceae. These are large bacterial families with both pathogenic and commensal bacteria members [75–77]. Although the exact mechanism of microbe-host interaction is not yet clear, the enriched relative abundance of Lachnospiraceae was shown to be positively correlated with low feed conversion ratio in pigs and gastrointestinal diseases and intestinal inflammation in human [78, 79]. Members of Bifidobacteriaceae, such as Bifidobacterium genus, are lactic acid producing bacteria that colonize into the intestine of pigs and human in their early life and are generally considered beneficial bacteria [80, 81]. The exact mechanism for this reduction remains unclear and needs to be further evaluated.

## Conclusions

Results from the present study demonstrated that supplementation of *Bacillus subtilis* DSM 32540 alleviated the severity of diarrhea caused by ETEC F18 infection and enhanced growth performance of weaned pigs. The enhanced disease resistance is highly correlated with lighter systemic and intestinal inflammation and better gut integrity in pigs supplemented with *Bacillus subtilis* DSM 32540. Thus, more nutrients and energy were used for growth instead of against bacterial infection in these pigs. Although *Bacillus pumilus* DSM 32539 was able to alleviate systemic inflammation, it had limited effects on growth performance and disease resistance of ETEC F18 challenged weaned pigs. Future research will consider incorporating metagenomics to provide more insight into the effects of *Bacillus subtilis* DSM 32540 on pigs’ gut microbial community. Large-scale animal trials are recommended to further evaluate the impacts of both *Bacillus* strains on performance of weaned pigs under commercial practice conditions.

## Supplementary information

**Additional file 1: Supplementary Table 1.** Gene-specific primer sequences and PCR conditions^1^. ^1^Thermal cycling conditions were 95 °C for 20 s and 95 °C for 1 s, followed by 40 cycles with 20 s at 60 °C. ^2^*CLDN1* = Claudin 1; *GAPDH* = Glyceraldehyde 3-phophate dehydrogenase; *IL1B* = Interleukin 1 beta; *IL6* = Interleukin 6; *MUC2* = Mucin 2; *OCLN* = Occludin; *PTGS2* = Cyclooxygenase 2; *TNF* = Tumor necrosis factor alpha; *ZO-1* = Zonula occludens-1. ^3^Accession number in GenBank database. **Supplementary Table 2.** Red blood cell profiles in enterotoxigenic *E. coli* F18 challenged pigs fed diets supplemented with probiotics ^a,b^Means without a common superscript are different (*P* < 0.05). ^1^RBC = red blood cell; HGB = hemoglobin; HCT = packed cell volume; MCV = mean corpuscular volume; MCH = mean corpuscular hemoglobin; MCHC = mean corpuscular hemoglobin concentration; RDW = red cell distribution width; MPV = mean platelet volume. Each least squares mean represents 8–10 observations. ^2^fL = femtolitre (10^− 15^ L). ^3^PRO1 = *Bacillus subtilis* DSM 32540. ^4^PRO2 = *Bacillus pumilus* DSM 32539. **Supplementary Table 3.** The relative abundance (%) of top four enriched families in different segments of the intestine of enterotoxigenic *E. coli* F18 challenged pigs fed diets supplemented with probiotics. ^a-e^Means without a common superscript are different (*P* < 0.05). Each least squares mean represents 8–10 observations. ^1^PRO1 = *Bacillus subtilis* DSM 32540. ^2^PRO2 = *Bacillus pumilus* DSM 32539.

**Additional file 2: Supplementary Figure 1.** Stacked bar plot showing the relative abundance of the family of Firmicutes (A), Actinobacteria (B), Proteobacteria (C), and Bacteroidetes (D) in the jejunum, ileum, and distal colon of enterotoxigenic *E. coli* F18 challenged pigs fed diets supplemented with probiotics on d 21 post inoculation. Each least squares mean represents 7–10 observations. PRO1 = *Bacillus subtilis* DSM 32540; PRO2 = *Bacillus pumilus* DSM 32539. **Supplementary Figure 2.** The rarefaction curves of 16S rRNA sequence data.

## Data Availability

All data generated or analyzed during this study are available from the corresponding author upon reasonable request.

## Abbreviations

ACTB: Beta-actin
ADFI: Average daily feed intake
ADG: Average daily gain
BW: Body weight
CLDN1: Claudin 1
*E. coli*: *Escherichia coli*
ETEC: Enterotoxigenic *Escherichia coli*
GAPDH: Glyceraldehyde 3-phosphate dehydrogenase
IL: Interleukin
LT: Heat-labile toxin
mRNA: Messenger RNA
MUC2: Mucin 2
OCLN: Occludin
PBS: Phosphate buffer saline
PI: Post-inoculation
PTGS2: Cyclooxygenase 2
ST: Heat-stable toxin
TNF: Tumor necrosis factor alpha
ZO1: Zona occludens-1
