# Supplementary material for: Dietary Bacillus spp. enhanced growth and disease resistance of weaned pigs by modulating intestinal microbiota and systemic immunity
Source: J Anim Sci Biotechnol. 2020 Sep 15;11:101. doi: 10.1186/s40104-020-00498-3 (PMC7491085; doi:10.1186/s40104-020-00498-3)
Supplement: Supplementary file 1 — Additional file 1: Supplementary Table 1. Gene-specific primer sequences and PCR conditions1. 1Thermal cycling conditions were 95 °C for 20 s and 95 °C for 1 s, followed by 40 cycles with 20 s at 60 °C. 2CLDN1 = Claudin 1; GAPDH = Glyceraldehyde 3-phophate dehydrogenase; IL1B = Interleukin 1 beta; IL6 = Interleukin 6; MUC2 = Mucin 2; OCLN = Occludin; PTGS2 = Cyclooxygenase 2; TNF = Tumor necrosis factor alpha; ZO-1 = Zonula occludens-1. 3Accession number in GenBank database. Supplementary Table 2. Red blood cell profiles in enterotoxigenic E. coli F18 challenged pigs fed diets supplemented with probiotics a,bMeans without a common superscript are different (P < 0.05). 1RBC = red blood cell; HGB = hemoglobin; HCT = packed cell volume; MCV = mean corpuscular volume; MCH = mean corpuscular hemoglobin; MCHC = mean corpuscular hemoglobin concentration; RDW = red cell distribution width; MPV = mean platelet volume. Each least squares mean represents 8–10 observations. 2fL = femtolitre (10− 15 L). 3PRO1 = Bacillus subtilis DSM 32540. 4PRO2 = Bacillus pumilus DSM 32539. Supplementary Table 3. The relative abundance (%) of top four enriched families in different segments of the intestine of enterotoxigenic E. coli F18 challenged pigs fed diets supplemented with probiotics. a-eMeans without a common superscript are different (P < 0.05). Each least squares mean represents 8–10 observations. 1PRO1 = Bacillus subtilis DSM 32540. 2PRO2 = Bacillus pumilus DSM 32539. [file 40104_2020_498_MOESM1_ESM.docx]

**Supplementary Table 1**. Gene-specific primer sequences and PCR conditions^1^

| Gene^2^ | Acc. No^3^ | Forward primer (5’ →3’) | Reverse primer (5’ →3’) |
| --- | --- | --- | --- |
| *ACTB* | DQ452569 | CGAGGCCCAGAGCAAGAG | TCCATGTCGTCCCAGTTGGT |
| *CLDN1* | NM001244539 | TCTTAGTTGCCACAGCATGG | CCAGTGAAGAGAGCCTGACC |
| *GAPDH* | [DQ845173](http://www.sciencedirect.com/science?_ob=RedirectURL&_method=externObjLink&_locator=genbank&_issn=03784274&_origin=article&_zone=art_page&_plusSign=%2B&_targetURL=http%253A%252F%252Fwww.ncbi.nlm.nih.gov%252Fentrez%252Fquery.fcgi%253Fcmd%253Dsearch%2526db%253Dnucleotide%2526doptcmdl%253Dgenbank%2526term%253DNM_214353%5baccn%5d) | ATAAGTGTGACTGCACCCGAAC | GGTGGGCTATCAATCAGATGTG |
| *IL1B* | NM 214055.1 | CCTTGAAACGTGCAATGATG | TTCAAGTCCCCTGTGAGGAG |
| *IL6* | AB194100.1 | CTGGCAGAAAACAACCTGAACC | TGATTCTCATCAAGCAGGTCTCC |
| *MUC2* | AK231524.1 | CAACGGCCTCTCCTTCTCTGT | GCCACACTGGCCCTTTGT |
| *OCLN* | NM001163647 | CAGCCAACGGGAAGATTCTG | ATGGCTTCCAGGTCGTCAT |
| *PTGS2* | [AF207824.1](https://www.ncbi.nlm.nih.gov/nucleotide/AF207824.1?report=genbank&log$=nucltop&blast_rank=3&RID=4RV9ZEVM014) | ATAAGTGTGACTGCACCCGAAC | GGTGGGCTATCAATCAGATGTG |
| *TNF* | EU682384.1 | AACCTCAGATAAGCCCGTCG | ACCACCAGCTGGTTGTCTTT |
| *ZO-1* | AJ318101 | CCGCCTCCTGAGTTTGATAG | CAGCTTTAGGCACTGTGCTG |

^1^Thermal cycling conditions were 95°C for 20 s and 95°C for 1 s, followed by 40 cycles with 20 s at 60°C.

^2^*CLDN1* = Claudin 1; *GAPDH* = Glyceraldehyde 3-phophate dehydrogenase; *IL1B* = Interleukin 1 beta; *IL6* = Interleukin 6; *MUC2* = Mucin 2; *OCLN* = Occludin; *PTGS2* = Cyclooxygenase 2; *TNF* = Tumor necrosis factor alpha; *ZO-1* = Zonula occludens-1.

^3^Accession number in GenBank database.

**Supplementary Table 2.** Red blood cell profiles in enterotoxigenic *E. coli* F18 challenged pigs fed diets supplemented with probiotics

| Item^1^ | Control | PRO1^3^ | PRO2^4^ | SEM | *P*-value |
| --- | --- | --- | --- | --- | --- |
| D 0 before inoculation |  |  |  |  |  |
| RBC, 10^6^/μL | 7.63 | 7.72 | 7.48 | 0.2 | 0.77 |
| HGB, g/dL | 10.23 | 10.18 | 9.88 | 0.23 | 0.57 |
| HCT, % | 31.16 | 30.74 | 30.39 | 0.64 | 0.72 |
| MCV, fL^2^ | 41.06 | 39.81 | 41 | 0.73 | 0.36 |
| MCH, pg | 13.5 | 13.21 | 13.33 | 0.34 | 0.79 |
| MCHC, g/dL | 32.86 | 33.11 | 32.48 | 0.51 | 0.71 |
| RDW, % | 22.96 | 23.47 | 25.2 | 0.81 | 0.23 |
| Platelets, 10^3^/μL | 402.7 | 385.4 | 430.3 | 36.98 | 0.75 |
| MPV, fL^2^ | 9.52 | 10.15 | 9.65 | 0.49 | 0.60 |
| Total protein, g/dL | 4.86 | 4.68 | 5.1 | 0.16 | 0.27 |
| D 3 post inoculation |  |  |  |  |  |
| RBC, 10^6^/μL | 7.31 | 7.27 | 7.3 | 0.23 | 0.99 |
| HGB, g/dL | 10.45 | 10.05 | 9.89 | 0.27 | 0.26 |
| HCT, % | 30.61 | 29.13 | 30.13 | 0.86 | 0.46 |
| MCV, fL^2^ | 41.91 | 40.17 | 41.39 | 0.61 | 0.18 |
| MCH, pg | 14.28 | 13.79 | 13.51 | 0.3 | 0.27 |
| MCHC, g/dL | 34.10^ab^ | 34.27^a^ | 32.75^b^ | 0.55 | <0.05 |
| RDW, % | 23.58^b^ | 24.25^ab^ | 26.14^a^ | 0.61 | <0.05 |
| Platelets, 10^3^/μL | 441.5 | 459.1 | 440.7 | 33.19 | 0.86 |
| MPV, fL^2^ | 10.89 | 10.53 | 9.93 | 0.39 | 0.25 |
| Total protein, g/dL | 5.01^b^ | 4.94^b^ | 5.59^a^ | 0.15 | <0.05 |
| D 6 post inoculation |  |  |  |  |  |
| RBC, 10^6^/μL | 7.72 | 7.01 | 7.46 | 0.22 | 0.14 |
| HGB, g/dL | 10.88^a^ | 9.71^b^ | 10.37^ab^ | 0.28 | <0.05 |
| HCT, % | 31.60^a^ | 28.25^b^ | 31.14^a^ | 0.67 | <0.05 |
| MCV, fL^2^ | 41.11 | 40.41 | 41.81 | 0.64 | 0.40 |
| MCH, pg | 14.16 | 13.9 | 13.9 | 0.29 | 0.88 |
| MCHC, g/dL | 34.42 | 34.41 | 33.27 | 0.49 | 0.28 |
| RDW, % | 23.91^b^ | 24.54^ab^ | 25.91^a^ | 0.67 | <0.05 |
| Platelets, 10^3^/μL | 411.2 | 419.7 | 455.3 | 40.15 | 0.74 |
| MPV, fL^2^ | 10.15 | 10.05 | 9.84 | 0.4 | 0.84 |
| Total protein, g/dL | 5.26^a^ | 4.79^b^ | 5.47^a^ | 0.14 | <0.05 |
| D 13 post inoculation |  |  |  |  |  |
| RBC, 10^6^/μL | 6.91 | 6.37 | 6.53 | 0.21 | 0.24 |
| HGB, g/dL | 10.07^a^ | 9.38^b^ | 9.51^ab^ | 0.24 | 0.079 |
| HCT, % | 30.96^a^ | 28.60^b^ | 29.23^ab^ | 0.78 | 0.071 |
| MCV, fL^2^ | 45.02 | 44.85 | 45.29 | 0.75 | 0.91 |
| MCH, pg | 14.67 | 14.71 | 14.72 | 0.27 | 0.98 |
| MCHC, g/dL | 32.6 | 32.7 | 32.51 | 0.36 | 0.91 |
| RDW, % | 28.34 | 29.73 | 28.81 | 0.86 | 0.57 |
| Platelets, 10^3^/μL | 405.7^a^ | 310.5^b^ | 428.0^a^ | 29.77 | <0.05 |
| MPV, fL^2^ | 10.08 | 9.93 | 10.13 | 0.34 | 0.92 |
| Total protein, g/dL | 5.00^b^ | 5.00^b^ | 5.43^a^ | 0.1 | <0.05 |
| D 21 post inoculation |  |  |  |  |  |
| RBC, 10^6^/μL | 6.88 | 6.87 | 6.3 | 0.26 | 0.31 |
| HGB, g/dL | 10.23 | 10.22 | 9.34 | 0.35 | 0.24 |
| HCT, % | 31.55 | 31.19 | 28.64 | 1.07 | 0.23 |
| MCV, fL^2^ | 46.09 | 45.33 | 45.82 | 0.88 | 0.82 |
| MCH, pg | 14.96 | 14.88 | 14.94 | 0.32 | 0.98 |
| MCHC, g/dL | 32.46 | 32.74 | 32.61 | 0.33 | 0.84 |
| RDW, % | 25.26 | 24.83 | 25.01 | 0.44 | 0.81 |
| Platelets, 10^3^/μL | 323.3 | 363.1 | 225.3 | 47.83 | 0.23 |
| MPV, fL^2^ | 9.44^ab^ | 10.20^a^ | 8.17^b^ | 0.43 | <0.05 |
| Total protein, g/dL | 5.01 | 5.3 | 5.3 | 0.1 | 0.14 |

^a,b^Means without a common superscript are different (*P* < 0.05).

^1^RBC = red blood cell; HGB = hemoglobin; HCT = packed cell volume; MCV = mean corpuscular volume; MCH = mean corpuscular hemoglobin; MCHC = mean corpuscular hemoglobin concentration; RDW = red cell distribution width; MPV = mean platelet volume. Each least squares mean represents 8-10 observations.

^2^fL= femtolitre (10^-15^ L).

^3^PRO1 = *Bacillus subtilis* DSM 32540.

^4^PRO2 = *Bacillus pumilus* DSM 32539.

**Supplementary Table 3**. The relative abundance (%) of top four enriched families in different segments of the intestine of enterotoxigenic *E. coli* F18 challenged pigs fed diets supplemented with probiotics

|  | **Jejunum** | | |  | **Ileum** | | |  | **Colon** | | |
| --- | --- | --- | --- | --- | --- | --- | --- | --- | --- | --- | --- |
|  | **Control** | **PRO1^1^** | **PRO2^2^** |  | **Control** | **PRO1** | **PRO2** |  | **Control** | **PRO1** | **PRO2** |
| Firmicutes |  |  |  |  |  |  |  |  |  |  |  |
| Erysipelotrichaceae | 0.44±0.50^cd^ | 0.29±0.42^d^ | 0.20±0.24^d^ |  | 2.50±3.93^bc^ | 0.40±0.46^d^ | 6.83±11.87^ab^ |  | 3.50±2.82^a^ | 2.29±2.07^ab^ | 1.98±2.17^ab^ |
| Lachnospiraceae | 1.31±1.15^b^ | 2.68±5.81^bc^ | 0.40±0.45^c^ |  | 0.36±0.56^c^ | 0.11±0.24^d^ | 0.15±0.18^cd^ |  | 16.94±6.66^a^ | 17.25±6.04^a^ | 20.28±6.14^a^ |
| Lactobacillaceae | 74.30±12.83^a^ | 61.82±23.88^ab^ | 72.56±31.50^a^ |  | 58.79±23.66^ab^ | 44.92±37.09^b^ | 57.14±31.44^ab^ |  | 15.23±4.16^c^ | 13.22±7.62^c^ | 10.81±11.27^c^ |
| Peptostreptococcaceae | 0.11±0.30^e^ | 0.16±0.38^de^ | 0.08±0.16^de^ |  | 2.89±3.16^ab^ | 0.59±0.78^cd^ | 5.73±5.61^a^ |  | 0.64±0.63^bc^ | 0,74±0.80^bc^ | 0.76±0.70^bc^ |
| Ruminococcaceae | 0.44±0.75^b^ | 0.13±0.29^bc^ | 0.12±0.20^c^ |  | 0.17±0.39^c^ | 0.08±0.17^c^ | 0.05±0.13^c^ |  | 10.62±3.62^a^ | 13.93±5.44^a^ | 16.51±6.65^a^ |
| Streptococcaceae | 6.50±11.57 | 19.23±19.92 | 10.53±20.18 |  | 22.48±25.44 | 46.71±37.96 | 17.81±14.27 |  | 9.79±16.29 | 15.46±15.23 | 3.88±7.91 |
| Veillonellaceae | 5.04±4.27^bc^ | 4.75±5.87^cd^ | 5.15±8.38^cde^ |  | 1.57±1.35^de^ | 0.72±1.12^e^ | 0.51±0.44^e^ |  | 12.44±5.93^a^ | 9.43±3.41^ab^ | 11.03±9.90^ab^ |
| Bacteroidetes |  |  |  |  |  |  |  |  |  |  |  |
| Muribaculaceae | 0.033±0.055^b^ | 0.011±0.019^bc^ | 0.059±0.157^bc^ |  | 0.025±0.054^bc^ | 0.000^c^ | 0.007±0.011^bc^ |  | 1.478±1.197^a^ | 2.849±4.040^a^ | 2.744±1.970^a^ |
| Prevotellaceae | 0.175±0.457^bc^ | 0.177±0.451^bc^ | 0.064±0.078^b^ |  | 0.137±0.299^bc^ | 0.008±0.015^c^ | 0.036±0.088^c^ |  | 9.659±4.760^a^ | 7.627±3.914^a^ | 10.273±4.410^a^ |
| Actinobacteria |  |  |  |  |  |  |  |  |  |  |  |
| Actinomycetaceae | 0.035±0.036 | 0.086±0.090 | 0.545±1.334 |  | 0.010±0.018 | 0 | 0.062±0.144 |  | 0 | 0 | 0 |
| Atopobiaceae | 1.272±1.424^a^ | 2.155±4.992^abc^ | 0.178±0.314^bcd^ |  | 0.367±0.412^bc^ | 0.049±0.156^d^ | 0.161±0.268^cd^ |  | 3.248±4.327^a^ | 3.840±9.770^ab^ | 0.346±0.465^bc^ |
| Bifidobacteriaceae | 7.369±4.607^a^ | 4.736±6.167^abc^ | 3.547±5.429^bcd^ |  | 5.856±4.969^ab^ | 1.674±2.479^cd^ | 4.137±7.194^bcd^ |  | 1.546±1.878^bcd^ | 2.179±3.626^cd^ | 0.710±.043^d^ |
| Coriobacteriaceae | 0.021±0.038^b^ | 0.018±0.037^b^ | 0.010±0.020^b^ |  | 0.009±0.021^b^ | 0.004±0.013^b^ | 0.000^b^ |  | 0.176±0.132^a^ | 0.285±0.155^a^ | 0.365±0.405^a^ |
| Micrococcaceae | 0.051±0.047^ab^ | 0.221±0.209^a^ | 0.574±1.326^ab^ |  | 0.033±0.045^abc^ | 0.049±0.113^bcd^ | 0.156±0.345^abc^ |  | 0.007±0.023^cd^ | 0.000^d^ | 0.000^d^ |
| Proteobacteria |  |  |  |  |  |  |  |  |  |  |  |
| Desulfovibrionaceae | 0.009±0.014^b^ | 0.000^c^ | 0.006±0.011^bc^ |  | 0.000^c^ | 0.000^c^ | 0.000^c^ |  | 0.227±0.188^a^ | 0.344±0.333^a^ | 0.179±0.113^a^ |
| Enterobacteriaceae | 0.137±0.146^ab^ | 0.095±0.095^abc^ | 0.426±0.717^a^ |  | 0.213±0.500^abcd^ | 0.078±0.140^bcde^ | 0.055±0.069^abcde^ |  | 1.907±6.017^cde^ | 0.008±0.017^de^ | 0.001±0.002^e^ |
| Pasteurellaceae | 0.209±0.351^ab^ | 1.888±2.636^ab^ | 0.022±0.059^c^ |  | 2.621±5.154^a^ | 2.799±7.350^bc^ | 4.911±7.889^a^ |  | 0.007±0.023^c^ | 0.000^c^ | 0.000^c^ |
| Succinivibrionaceae | 0.080±0.099^b^ | 0.018±0.021^bcd^ | 0.034±0.040^bc^ |  | 0.015±0.030^cde^ | 0.009±0.029^de^ | 0.000^e^ |  | 5.154±5.426^a^ | 3.053±3.441^a^ | 4.710±4.871^a^ |

^a-e^Means without a common superscript are different (*P* < 0.05). Each least squares mean represents 8-10 observations.

^1^PRO1 = *Bacillus subtilis* DSM 32540.

^2^PRO2 = *Bacillus pumilus* DSM 32539.
