# Supplementary material for: Dietary Bacillus spp. enhanced growth and disease resistance of weaned pigs by modulating intestinal microbiota and systemic immunity
Source: J Anim Sci Biotechnol. 2020 Sep 15;11:101. doi: 10.1186/s40104-020-00498-3 (PMC7491085; doi:10.1186/s40104-020-00498-3)
Supplement: Supplementary file 2 — Additional file 2: Supplementary Figure 1. Stacked bar plot showing the relative abundance of the family of Firmicutes (A), Actinobacteria (B), Proteobacteria (C), and Bacteroidetes (D) in the jejunum, ileum, and distal colon of enterotoxigenic E. coli F18 challenged pigs fed diets supplemented with probiotics on d 21 post inoculation. Each least squares mean represents 7–10 observations. PRO1 = Bacillus subtilis DSM 32540; PRO2 = Bacillus pumilus DSM 32539. Supplementary Figure 2. The rarefaction curves of 16S rRNA sequence data. [file 40104_2020_498_MOESM2_ESM.docx]

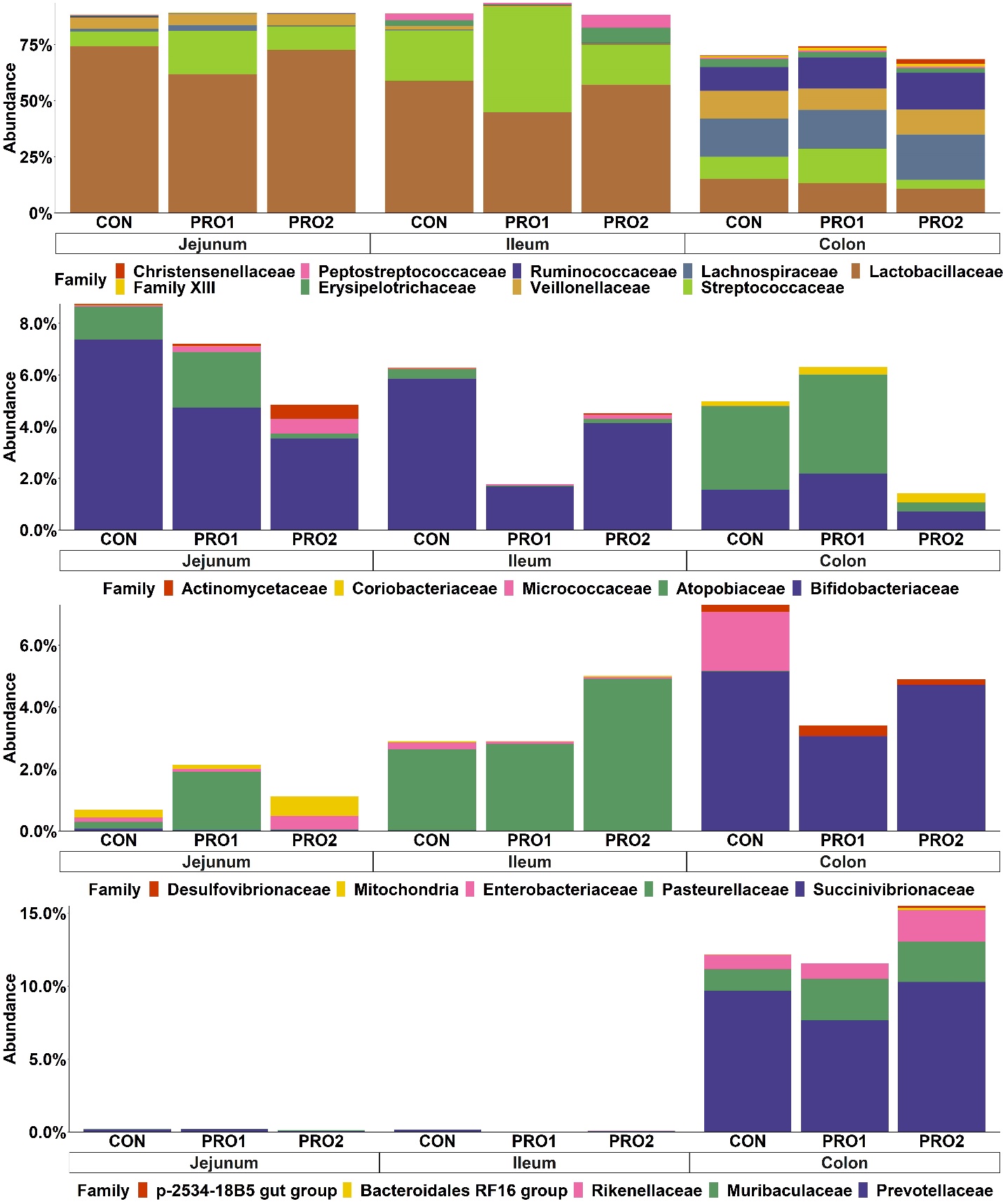


**Supplementary Figure 1.** Stacked bar plot showing the relative abundance of the family of Firmicutes (A), Actinobacteria (B), Proteobacteria (C), and Bacteroidetes (D) in the jejunum, ileum, and distal colon of enterotoxigenic *E. coli* F18 challenged pigs fed diets supplemented with probiotics on d 21 post inoculation. Each least squares mean represents 7-10 observations. PRO1 = *Bacillus subtilis* DSM 32540; PRO2 = *Bacillus pumilus* DSM 32539.


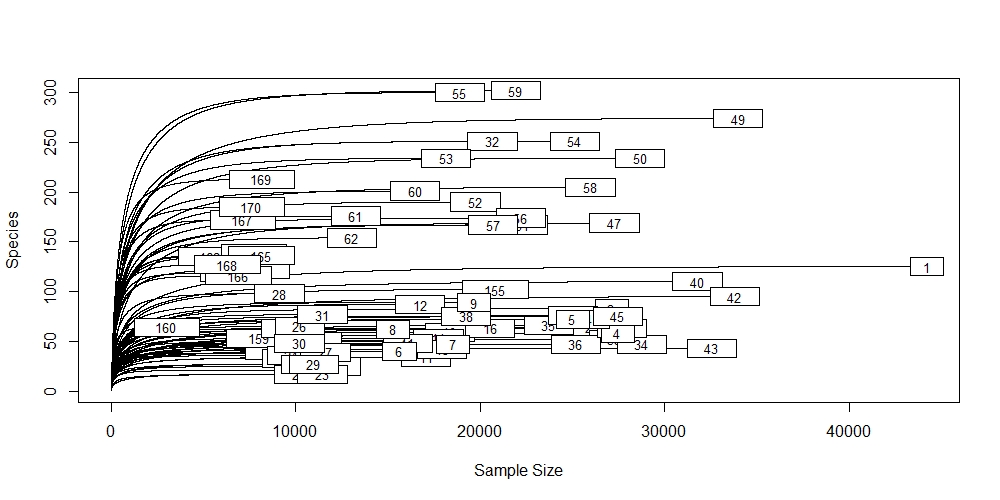


**Supplementary Figure 2**. The rarefaction curves of 16S rRNA sequence data.
